# Comparison of genomes and proteomes of four whole genome-sequenced *Campylobacter jejuni* from different phylogenetic backgrounds

**DOI:** 10.1371/journal.pone.0190836

**Published:** 2018-01-02

**Authors:** Clifford G. Clark, Chih-yu Chen, Chrystal Berry, Matthew Walker, Stuart J. McCorrister, Patrick M. Chong, Garrett R. Westmacott

**Affiliations:** 1 Division of Enteric Diseases, National Microbiology Laboratory, Public Health Agency of Canada, Winnipeg, Manitoba, Canada; 2 Mass Spectrometry and Proteomics Core Facility, National Microbiology Laboratory, Public Health Agency of Canada, Winnipeg, Manitoba, Canada; 3 Bioinformatics Core, National Microbiology Laboratory, Public Health Agency of Canada, Winnipeg, Manitoba, Canada; Institut National de la Recherche Agronomique, FRANCE

## Abstract

Whole genome sequencing (WGS) has been used to assess the phylogenetic relationships, virulence and metabolic differences, and the relationship between gene carriage and host or niche differentiation among populations of *C*. *jejuni* isolates. We previously characterized the presence and expression of CJIE4 prophage proteins in four *C*. *jejuni* isolates using WGS and comparative proteomics analysis, but the isolates were not assessed further. In this study we compare the closed, finished genome sequences of these isolates to the total proteome. Genomes of the four isolates differ in phage content and location, plasmid content, capsular polysaccharide biosynthesis loci, a type VI secretion system, orientation of the ~92 kb invertible element, and allelic differences. Proteins with 99% sequence identity can be differentiated using isobaric tags for relative and absolute quantification (iTRAQ) comparative proteomic methods. GO enrichment analysis and the type of artefacts produced in comparative proteomic analysis depend on whether proteins are encoded in only one isolate or common to all isolates, whether different isolates have different alleles of the proteins analyzed, whether conserved and variable regions are both present in the protein group analyzed, and on how the analysis is done. Several proteins encoded by genes with very high levels of sequence identity in all four isolates exhibited preferentially higher protein expression in only one of the four isolates, suggesting differential regulation among the isolates. It is possible to analyze comparative protein expression in more distantly related isolates in the context of WGS data, though the results are more complex to interpret than when isolates are clonal or very closely related. Comparative proteomic analysis produced log_2_ fold expression data suggestive of regulatory differences among isolates, indicating that it may be useful as a hypothesis generation exercise to identify regulated proteins and regulatory pathways for more detailed analysis.

## Introduction

*Campylobacter jejuni* is a fascinating human pathogen for which we have a rapidly growing, but still incomplete, understanding of virulence, host adaptation, environmental niche, and transmission through water and the food chain [1]. *Campylobacter* spp. are important human pathogens globally [2–5] and *C*. *jejuni* is capable of causing serious or disruptive sequelae such as the Guillain-Barré Syndrome (GBS), irritable bowel syndrome, and reactive arthritis [2,6,7]. Poultry, unpasteurized milk, water, and domesticated animals are common sources of human infection, while travel is an important risk factor [2].

*C*. *jejuni* genomes are relatively small, around 1.7 Mb, with well-defined hypervariable regions associated with capsular polysaccharide biosynthesis, lipooligosaccharide (LOS) biosynthesis, and flagellar glycosylation, as well as prophages [8,9]. There is a high rate of recombination due to horizontal gene transfer (HGT) resulting in variability of allelic diversity across *C*. *jejuni* genomes and varying degrees of disruption of the overall clonal population structure, such that the simultaneous introduction of large numbers of polymorphisms by HGT can rapidly generate novel phenotypes [10]. Comparative genomics studies characterize genomic differences and correlate them with the phylogeny and phenotypes of the organisms or lineages under study [11–13]. This approach has been used to confirm that *C*. *jejuni* and *C*. *coli* are separate species [14], investigate the genomic biology related to invasiveness of *C*. *jejuni* clonal complex (CC) ST-667 [15], and detect markers associated with niche differentiation, host adaptation, and host attribution [16–18].

While comparative genomics can identify sets of genes that display variability among isolates, characterizing proteins and proteomes is essential for understanding how the genotype determines the phenotype of organisms [19]. Shotgun proteomics using isobaric tags, as in the isobaric tags for relative and absolute quantification (iTRAQ) methods, allows highly accurate quantitation of multiple samples in a single measurement [20]. Most studies that deal with characterization of *C*. *jejuni* proteomes have assessed a single isolate or strain using different growth conditions or times or two isogenic or nearly isogenic isolates [21–24]. 8-plex iTRAQ comparison of an *E*. *coli* K12 *relE* mutant vs wild type in chemostat cultures was used to identify up- and down-regulated proteins and map them to regulatory networks [25]. Multi-omics approaches, including comparative genomic analysis, transcriptomics, and proteomics, have been used to elucidate the biology underlying hypervirulence of *C*. *jejuni* clone SA [26] and to validate the genome of a *C*. *concisus* strain [27]. Changes in protein expression levels of *C*. *jejuni* 81–176 after infection of COS-1 cells were assessed using a comparative proteomics approach combining in-gel digestion with LC-MS/MS [28], and iTRAQ comparative proteomic analysis has been employed to detect proteins differentially expressed after colonization of chickens [29]. A label-free MS/MS technology was used to characterize the regulation of *C*. *jejuni* NCTC 11168 proteins in response to porcine mucin [30]. iTRAQ and label-free proteomics methods have also been used to compare the proteome and glycoproteome as part of a multi-omics study to characterize differences between clinical and laboratory-adapted variants of NCTC 11168 [31]. These studies demonstrate the utility of proteomics approaches in general, and of iTRAQ proteomics approach specifically, in assessing regulation of protein expression.

In comparative proteomics experiments using labeled peptides the relative ratios of peptide intensities are used to calculate corresponding protein-level ratios, typically using peptides that are unique to each protein (tryptic peptides only in one protein in the database used for searching, termed ‘exclusive peptides’ herein). Inclusion of shared peptides present in more than one protein (non-exclusive peptides) may introduce significant quantification errors [32], but may also be useful for some analysis.

Acquiring whole genome sequence (WGS) alone is insufficient for understanding the biology of the organisms; additional omics investigations are required. We previously used 4-plex iTRAQ proteomics to detect expression of proteins associated with the CJIE4 prophage in *C*. *jejuni* in isolates with different serotypes and multi-locus sequence types (STs) [33]. The main hypotheses underlying the current work is that we can specifically identify changes in protein expression that may be a consequence of regulatory differences between isolates as well as identify those protein expression changes that differentiate proteins encoded by genes that are unique to each isolate or serotype. We also wanted to use the comparative proteomic results acquired in the study to illuminate differences in protein expression results when exclusive and non-exclusive peptides are used for the analysis of *C*. *jejuni* proteomes.

Complete, finished genomes of the four isolates used in the study were compared with comparative proteomic data. Results indicate that 1) there are protein expression differences among isolates that may be due to regulatory differences, 2) proteins unique to an isolate are clearly identified as such in 4-plex iTRAQ proteomics experiments, and 3) alleles of proteins encoded by genes with 99% identity are frequently distinguished, complicating data analysis. When results using exclusive and non-exclusive peptides are used for analysis of comparative proteomic data different artefacts associated with each set of results further complicates interpretation.

## Methods

### Strains and growth conditions

The four isolates selected for whole genome sequencing and comparative genomic analysis were associated with the investigation into the spring 2000 *Campylobacter* and *E*. *coli* outbreak in Walkerton, Ontario, Canada, though they were not associated with the outbreak itself [34]. Isolates 00–0949 and 01–1512 were both heat stable (HS, Penner) serotype HS:2, multi-locus sequence type (MLST) ST8, and flagellar short variable region (fla-SVR) sequence type 356, but had different pulsed-gel electrophoresis (PFGE) patterns. 00–0949 was isolated from a human source in Québec in 2000, while 01–1512 was isolated from a human in New Brunswick in 2000. Isolate 00–6200 was obtained from a human in Walkerton, Ontario in 2000, but did not appear to be part of the outbreak. It was serotype HS:O4,13 with ST806 and fla-SVR type 41. Finally, isolate 00–1597 was isolated from a human in Alberta in 2000 and was HS:9,37 with ST930 and fla-SVR 9. *C*. *jejuni* isolates were kept for long-term storage in either 20% skim milk or glycerol peptone water (25% v/v glycerol, 10 g/L neopeptone, 5 g/L NaCl) at -80°C. For use, *C*. *jejuni* isolates with a low passage number were retrieved from storage at -80°C, plated to Oxoid Mueller-Hinton agar (Oxoid Inc.) containing 10% sheep red blood cells (OMHA + blood), and grown for 48 h at 37°C under a microaerobic atmosphere (5% O_2_, 10% CO_2_, 85% N_2_) in anaerobic jars after twice replacing air with the gas mixture.

### Genome sequencing, assembly, closure, and annotation

#### Sequencing

Genomic DNA was prepared from isolates cultured overnight at 42°C on OMHA + blood using Epicentre Metagenomic DNA Isolation kits for Water (Illumina) according to the manufacturer’s instructions. DNA was quantitated using Qubit dsDNA BR assay kits (Life Technologies, Invitrogen). Sample libraries were prepared using a MiSeq Nextera^®^ XT DNA library preparation kit (Illumina). Whole genome sequencing was performed by 250 bp paired-end read sequencing on the Illumina MiSeq sequencer using the MiSeq^®^ Reagent Kit V2 and 500 cycles on the Illumina MiSeq platform to obtain an average genome coverage of 30–50×. Sequence reads were assembled into contigs using the SPAdes assembler (v3.0) [35]. Contigs smaller than 1kb and with average genome coverage less than 15× were filtered and removed from the analysis. The remaining contigs were closed and finished using Staden gap v4.10 by read mapping to the reference genome, NCTC11168, plus a combination of PCR and Sanger sequencing for gap closure. Fasta files for each genome were sent to Genomes (NCBI) for annotation using the NCBI prokaryotic annotation pipeline.

#### Large-scale blast score ratio (LS-BSR) analysis

LS-BSR analysis [36] was carried out using DNA fasta files from the NCBI annotated sequences for each isolate, resulting in a file of centroids and associated protein sequences. Proteins were annotated using BLAST and the resulting datasets used for comparisons of genomic and proteomic data.

#### iTRAQ comparative proteomics analysis

The protocol for 4-plex iTRAQ proteomic analysis of the four *C*. *jejuni* isolates was carried out as previously described [22]. The four isolates were recovered from -80°C frozen stocks as outlined above. For each experimental replicate each isolate was subcultured to two plates of Oxoid Mueller-Hinton agar and grown for 48h at 37°C under microareobic conditions. Experimental replicates were done on three different days using freshly subcultured bacteria; three replicates were done over the course of two weeks, and the process was repeated 11 months later to provide a total of six replicate experiments. Growth was harvested in PBS, pH 7.0–7.2 (Gibco; Invitrogen) and bacteria were collected in 1.5 ml screw-capped Eppendorf tubes, lysed with acid-washed 212–300 μm glass beads (Sigma-Aldrich Canada Ltd., Oakville, ON), and boiled. Proteins were extracted by “bead beating” for 5 min at low speed on a Genie 2 vortex mixer fitted with a 12 place Ambion Vortexer Adapter for Genie 2 Vortex Mixer attachment (Applied Biosystems Canada, Mississauga, ON). The protein concentration of each preparation was estimated using a Pierce Protein Assay kit (Fisher Scientific, Whitby, ON) according to the manufacturer’s protocol. Protein preparations were used immediately or stored at -80°C for up to two months.

#### Protein modification and digestion

To minimize variability each set of three replicate experiments was subjected to protein modification and labelling at the same time. Crude protein suspensions containing 100 μg protein were dried and suspended in 50 μl of freshly made SDS solubilization buffer (4% SDS, 50 mM HEPES buffer pH 8.3, 100 mM DTT), heated at 95°C for 5 min, and placed in a -20°C freezer overnight. After addition of 350 μl fresh Urea Exchange Buffer (UEB; 8M urea in 50 mM HEPES, pH 8.3) to the samples, the buffer was removed using Nanosep 10K spin filter cartridges (VWR International LLC, Mississauga, ON) and centrifuged at 10,000 × g. Samples were exchanged twice with fresh UEB followed by centrifugation, alkylated by adding 50 mM iodoacetamide (IAA Reagent, Sigma-Aldrich) in UEB, then exchanged a further three times with UEB. The buffer was then exchanged twice by replacement in spin columns with 150 μl of 50 mM HEPES, pH 8.3. DNA was removed by the addition of 50 μl of freshly made Benzonase (Sigma-Aldrich) solution (20 U/μl Benzonase in 42 mM HEPES, pH 8.3 containing 2 mM MgCl_2_) and spin columns containing the sample exchanged three times with 100 μl of 50 mM HEPES, pH 8.3 followed by centrifugation. Proteins were digested ON at 37°C with 5 μg trypsin (Trypsin Gold, mass spectrometry grade, Promega) dissolved in 5 μl of100 μl of 0.1% formic acid (vol/vol) added just before use to 45 μl of 50 mM HEPES, pH 8.3. Peptides were recovered from the cartridge by centrifugation using 50 mM HEPES, pH 8.3 for recovery.

#### iTRAQ labelling

Peptides were dried and suspended in 30 μl 100 mM HEPES, pH 8.3. 70 μl of 100% ethanol was added to each iTRAQ label and mixed. Each iTRAQ label solution was then added to a peptide preparation, mixed, collected by centrifugation, and incubated ON at RT. The next day sterile MilliQ water was added to quench the labelling reaction and the mixtures were dried. These mixtures were stored at -20°C until the next step. Labelled peptides were thawed, dissolved in 40 μl water, vortexed, and centrifuged to remove insoluble material. After removal of supernatant to a fresh tube, 1 μl of each labelled peptide mix was added to 56 μl nano LC buffer A (2% acetonitrile, 0.1% formic acid) in a 300 μl PTFE vial and analyzed by nano liquid chromatography as outlined below. Results of the analysis were used to accurately normalize the amount of each labelled peptide mixture for chromatography and mass spectrometry. Labelled peptide fractions were mixed to provide the same amount of peptide from each of the four isolates from each replicate experiment using approximately 10 μl volumes of each labelled peptide.

#### Liquid chromatography and mass spectrometry

iTRAQ-labelled tryptic peptide samples (100 μg) were fractionated by high-pH, C_18_-reversed phase liquid. Mixed peptides were dried and suspended in LC buffer A (20 mM ammonium formate, pH 10), then resolved by a gradient of LC buffer A and buffer B (20 mM ammonium formate and 90% acetonitrile, pH 10). Fractions were collected across the peptides elution profile (10–75 min), dried and resuspended in 40 μl of nano LC buffer A. Each fraction was separately analysed using a nano-flow Easy nLC II (Thermo Fisher Scientific) connected in-line to an LTQ Orbitrap Velos mass spectrometer (Thermo Fisher Scientific) with a nanoelectrospray ion source (Thermo Fisher Scientific). Data were acquired using a data-dependent method, dynamically choosing the top 10 abundant precursor ions from each survey scan for isolation in the LTQ and fragmentation by HCD at 45% normalized collision energy. The survey scans were acquired in the Orbitrap over *m/z* 300–1700 with a target resolution of 60,000 at *m/z* 400, and the subsequent fragment ion scans were acquired in the Orbitrap over a dynamic *m/z* range with a target resolution of 7500 at *m/z* 400. The lower threshold for selecting a precursor ion for fragmentation was 1000 counts. Dynamic exclusion was enabled using a list size of 500 features, a *m/z* tolerance of 15 ppm, a repeat count of 1, a repeat duration of 30 s, and an exclusion duration of 15 s, with early expiration disabled.

#### Data processing

All spectra were processed using Mascot Distiller v2.3.2 (Matrix Science), and database searching was done with Mascot v2.5.1 (Matrix Science). Searches were performed against databases constructed using protein fasta files for: 1) the 00–0949 genome, pTet plasmid, and pVir plasmid; 2) the 01–1512 genome, pTet plasmid pCj1, and pVir plasmid pCj2; 3) the 00–6200 genome; 4) the 00–1597 genome. The decoy database option was selected and the following parameters were used: carbamidomethylation (C) and iTRAQ (K and N-terminus) as fixed modifications, oxidations (M) as a variable modification, fragment ion mass tolerance of 0.5 Da, parent ion tolerance of 10 ppm, and trypsin enzyme with up to 1 missed cleavage. Mascot search results were imported into Scaffold Q+ v4.6.1 (Proteome Software) using intensity-based analysis (centroided peak intensity) with peptide thresholds of 0.1% false discovery rate (FDR) and protein thresholds of 1% FDR and at least 2 peptides per protein. Analysis was done both with and without inclusion of non-exclusive peptides where unique peptides are defined as those associated with a single protein group. Results were expressed as log_2_ ratios and downloaded as Excel files. Statistical comparisons of each pair of isolates were done within Scaffold using the Mann-Whitney test with Benjamini-Hochberg correction for multiple testing; the software also provided log_2_ ratio for each pairwise comparison (S1 Spreadsheet). Heat maps and principal component analysis (PCA) plots were generated using R3.3.2 [37] based on Excel Samples Report files from Scaffold Q+ v4.6.1 with the reference isolate set as 00–0949. The hierarchical clustering of heat maps was conducted using the complete linkage method on a dissimilarity matrix using a Euclidean distance measure with heatmap.2 function in R, and PCA was conducted using prcomp with variable zero-centered and scaled to have unit variance. Differential expressed protein counts were visualized using UpSet plots [38] generated with the UpSetR package [39] in R. GI numbers of identified proteins were converted to Uniprot ID using Retrieve/ID mapping (http://www.uniprot.org/uploadlists/), and GO enrichment analyses were conducted using Fisher’s exact test in Perseus [40].

## Results and discussion

**Fig 1:**
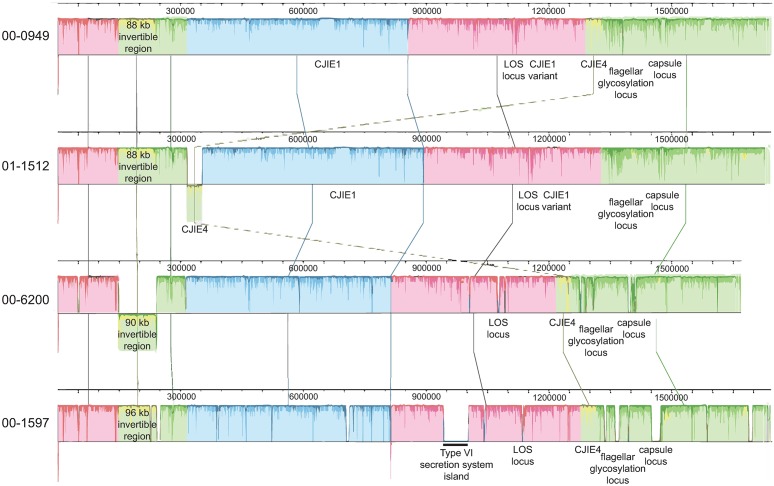
Alignment showing the genome organization of the four *C*. *jejuni* isolates used in this study. The figure was prepared using Progressive Mauve (Mauve 20150226 build 10 [44]).

**Fig 2:**
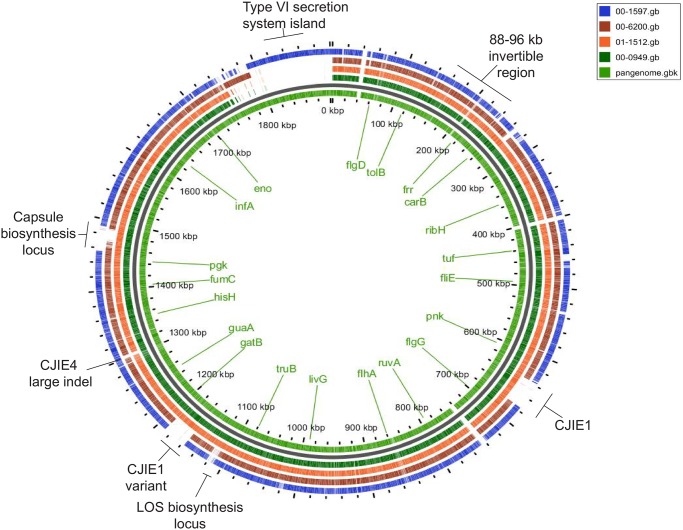
Comparison of *C*. *jejuni* isolate pangenome comparison. The BLAST atlas was obtained using GView Server [43] and further annotated using Adobe Illustrator.

The characteristics of the isolates used for the analysis and of the sequenced genomes are summarized in Table 1. *C*. *jejuni* isolates 00–0949 and 01–1512 are very similar to each other, with the same HS serotype, multilocus sequence type (MLST) designation (ST), clonal complex (CC), and similar numbers of genes and CDSs. They also have the same number and locations of poly G/C homopolymeric tracts, which differ in the other two isolates (S1 Table). Isolate 00–6200 is more divergent, having a different HS serotype and ST, but belonging to the same CC ST-21. The chromosome of this isolate (excluding plasmids) has fewer genes and CDSs than the chromosomes of the other two CC ST-21 isolates. It also has an inversion of the invertible region between Tlp proteins, which is a characteristic of *C*. *jejuni* isolates [41], compared with the 3 other isolates (Fig 1). The most divergent isolate was 00–1597, which had different HS, ST, and CC types. These isolates differ in the carriage of prophages, the presence of a Type VI secretion island containing a Type VI secretion system gene cluster, and the content of hypervariable regions (Figs 1 and 2). Of the 1984 ORFs/genes identified with LS-BSR [36] analysis of the 4 isolates, 626 have 100% nucleotide identity, 1500 (75.6%) are present in all isolates at a level of 90% identity or greater, 1556 (76.4%) are present at 80% identity or greater, and 1589 (80.1%) are present at 70% identity or greater. This corresponds to the extensive core genome identified using GView Server [42,43] (Fig 1 and S1 Fig). In contrast, the accessory genome of isolates 00–0949 and 01–1512 is more extensive than isolates 00–6200 and 00–1597, and appears to be almost identical in both 00–0949 and 01–1512 (S2 Fig). While neither isolate 00–0949 nor isolate 01–1512 harbored any unique genes when included in comparisons of all 4 isolates, a small number of unique genes were detected in isolate 00–6200 and a much larger number are found in isolate 00–1597, some of which correspond to large indels introduced into the genome (Fig 1 and S3 Fig). LS-BSR analysis indicates that 1734/1984 (87%) genes are identical in 00–0949 and 01–1512 while 1082/1984 00–6200 (55%) genes and 802/1984 (40%) 00–1597 genes are identical with those of both 00–0949 and 01–1512. Only 638/1984 (32%) genes are identical in both isolates 00–6200 and 00–1597. A majority of unique genes in isolate 00–6200 are associated with the capsular polysaccharide biosynthesis (CPB) gene cluster. Unique genes of isolate 00–1597 are associated a large type VI secretion system island containing a type VI secretion system gene cluster and the HS:9 CPB gene cluster. Because two HS:2 isolates were included in the experimental system and because the CJIE1, CJIE1variant, and CJIE4 prophages are found in more than one isolate, these two isolates did not carry identifiable unique genes.

**Table 1:** Summary data for the 4 complete sequenced isolates and genomes.

Characteristic	Isolate			
	00–0949	01–1512	00–6200	00–1597
HS (Penner) serotype	HS:2	HS:2	HS:4,13	HS:9,37
MLST sequence type (ST)	8	8	806	930
MLST clonal complex (CC)	ST-21	ST-21	ST-21	NA
Genes	1,974	1,971	1,755	1,817
CDS	1,884	1,885	1,673	1,721
Pseudo Genes	36	32	28	42
CRISPR arrays	1	1	1	1
rRNAs (5S, 16S, 23S)	3, 3, 3	3, 3, 3	3, 3, 3	3, 3, 3
Complete rRNAs (5S, 16S, 23S)	3, 3, 3	3, 3, 3	3, 3, 3	3, 3, 3
tRNAs	44	44	44	44
ncRNA	1	1	1	1
Frameshifted genes	25	22	26	33
Frameshifted genes on monomer runs	3	3	4	11
Frameshifted genes not on monomer runs	3	4	3	2
Plasmids	2	2	0	0
Size of invertible region (bp)	88,465	88,465	89,946	96,109

GenBank accession numbers for the isolate data in this table: 00–0949, NZ_CP010301; 00–1512, NZ_CP010072; 00–6200, NZ_CP010307, 00–1597, NZ_CP010306.

NA, not applicable, meaning the ST was not assigned to a clonal complex.

### Gene content and protein expression differ in the four isolates

There are 127 unique genes in 00–1597 absent (defined as having <70% nucleotide identity) in each of the other 3 isolates (Table 2). 4-plex iTRAQ comparative proteomics experiments (iTRAQ experiments) indicated proteins of 59 of these unique genes are not detected under the growth conditions used, while protein products of 60 unique genes can be detected only in isolate 00–1597 (ie. at very high log_2_ fold change values compared with other isolates) when data are analyzed in Scaffold using either exclusive or non-exclusive peptides. Eight genes are annotated as pseudogenes in the original GenBank submission (Genbank accession no. CP010301) used in protein databases for peptide identification with Mascot, and therefore are not included in iTRAQ results. In total, 73 genes are absent from isolate 00–1597 (≤ 70% nucleotide identity) but present in one or more of the other 3 isolates. This includes the PR2 hypervariable region responsible for fucose utilization [45] present between *rpoC* and *rpsL* in isolates 00–6200, 00–0949, and 01–1512 and absent at this location in isolate 00–1597. Within the PR2 hypervariable region of the other three isolates only the amidohydrolase, which is a pseudogene in all three isolates, and the hypothetical protein are not detected in iTRAQ experiments. With the exception of the transcriptional regulator/fucose operon repressor, all proteins encoded by PR2 are expressed at higher log_2_ fold change levels in isolate 00–6200 (S2 and S3 Spreadsheets). Fucose utilization is associated with enhanced growth, host colonization, chemotaxis toward fucose, and inhibition of biofilm formation [46,47]. The presence of the fucose utilization region appears to be stratified by *C*. *jejuni* clonal complex [46,48]. Isolate 00–1597 may therefore have different metabolic and virulence properties than the other three isolates.

**Table 2:** Distribution in genomes and expression of unique chromosomal genes of the 4 isolates.

Hypervariable region, genomic element, or prophage	00–0949 + 00–1512 (HS:2)		00–6200 (HS:4)		00–1597 (HS:9)	
	# unique genes	# proteins detected	# unique genes	# proteins detected	# unique genes	# proteins detected
CJIE1 + CJIE1 variant prophages	76	13	0	NA	0	NA
CJIE4 prophage	0	NA	2	1	0	NA
LOS biosynthesis gene cluster	5	3	0	NA	1	1
flagellar glycosylation gene cluster	1	1	1	1	3	1
capsule biosynthesis gene cluster	15	15	11	8	20	18
T6SS island, not T6SS gene cluster	0	0	0	0	32	6
T6SS gene cluster	0	0	0	0	13	9
putative invertible element	0	0	0	0	6	1
not in a defined genomic element	19	3	16	4	52	17
Total	115	34	30	14	127	53

NA, not applicable

An additional two genes provided further insights into protein detection in isolate 00–1597 (S2 and S3 Spreadsheets). Hypothetical protein PJ17_03955 is not detected when exclusive peptides are used to analyze comparative proteomics experiments. In contrast, when non-exclusive peptides are used the protein is detected and the log_2_ fold change values were higher for isolates 00–0949 and 01–1512 despite only 65% nucleotide identity between 00–1597 and both 00–0949 and 01–1512. Comparisons using Clustal Omega multiple sequence alignment and NCBI blastp (accessed Oct18, 2016) confirmed that the proteins found in HS:2 isolates 00–0949 and 01–1512 are identical but exhibit only 67% identity with the homolog in isolate 00–1597. Exclusive peptides are not detected, and inclusion of non-exclusive peptides leads to an artefactual comparison of the different proteins. A similar result was obtained for carbonic anhydrase (PJ17_02995).

Isolate 00–6200 harbors 30 genes not present in the other three isolates (Table 2). These are present within hypervariable regions, the CJIE4 prophage, and distributed throughout the genome. Proteins were detected for most unique CPB genes (see below), one unique flagellar glycosylation gene, one unique CJIE4 prophage gene, and four of the remaining unique genes. The ATPase AAA transporter (PJ18_00705), hypothetical protein (PJ18_05300), pyrimidine nucleotide-disulfide oxidoreductase (PJ18_05295), and spermidine dehydrogenase (PJ18_05730) are all much more highly expressed in isolate 00–6200 when both exclusive and non-exclusive peptides are included in the analysis (S2 and S3 Spreadsheets), consistent with the fact they are encoded by genes unique to this isolate. Three genes were annotated as pseudogenes in the original GenBank version (Genbank accession no. CP010307) used in protein databases for peptide identification with Mascot, and therefore are not detected in the comparative proteomics results. There are 14 genes absent from isolate 00–6200 (<70% identity) and present at ≥70% identity in all 3 remaining isolates. Three proteins were absent from isolate 00–6200 and detected in isolates 00–0949, 01–1512, and 00–1597. These were UDP-galactopyranose mutase, alpha-ketoglutarate permease, and an oxidoreductase.

Of the 115 genes common to the two HS:2 isolates 00–0949 and 01–1512 and present at <70% nucleotide identity in the other two isolates, most were associated with CJIE1 and CJEI1 variant prophages and hypervariable regions (Table 2). In addition, there are 29 gene differences between 00–0949 and 01–1512. Among these are 15 associated with the CJIE4 prophage (9 with the CJIE4 alternate indels discussed in previous work [33]). Three are associated with motility accessory factor proteins within the flagella glycosylation gene cluster, all of which are expressed. Two *tlp* genes encoding the transducer-like proteins (methyl-accepting chemotaxis proteins) Tlp4 and Tlp3 bracketing the putative invertible element are expressed and differ in the two isolates (PJ19_01250, disrupted in 00–0949, and PJ16_08505, respectively). A gene encoding nucleoside hydrolase (PJ18_01635) present and expressed in isolates 00–0949 and 00–6200 is disrupted and not expressed in isolate 01–1512. Finally, though UDP pyrophosphate phosphatase in isolate 00–0949 lacks 2/7 IDLNNTK motifs present in the same protein in 01–1512, both are expressed at the same level. Genes within the alternate CJIE4 indels are shared with one other isolate in all cases.

Isolates 00–0949 and 01–1512 both harbour two plasmids, pTet and pVir, that are not present in the other two isolates. Plasmid proteins are only detected in these isolates (S2 and S3 Spreadsheets). In addition to hypothetical proteins, tetracycline resistance protein TetM (PJ16_09570) and a toxin-antitoxin system protein (PJ16_09875) are detected at similar levels in both isolates. In contrast, Type IV secretion/competence protein VirB9 protein is detected at much higher log_2_ fold change values in 00–0949 (PJ16_09675) than 01–1512 (PJ19_09660) when both exclusive and non-exclusive peptides are used for data analysis.

**Fig 3:**
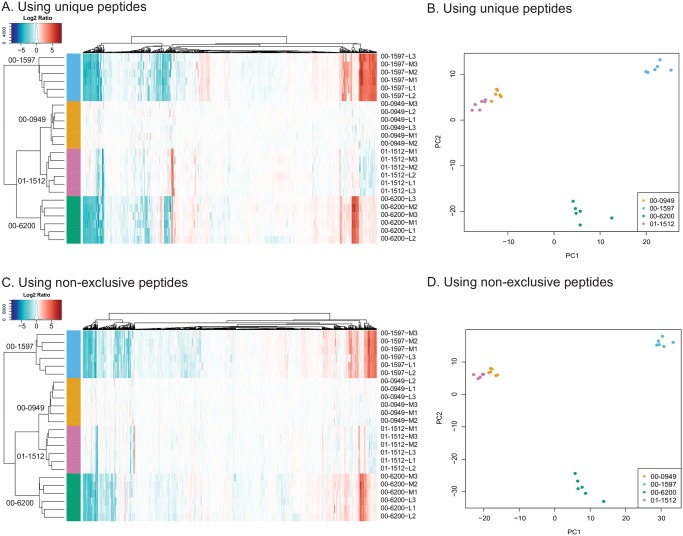
Detection of proteins from each of the four isolates. A. Heat map comparing relative log_2_ fold change values of proteins for each isolate using 00–0949 as the reference. Only exclusive peptides were used for analysis in Scaffold. The scale bar shows the relationship of color to log_2_ fold change values. Labels to the right of each lane identify the isolate and experimental replicate associated with that lane. B. PCA comparing the first two components when only exclusive peptides were used for analysis in Scaffold. C. Heat map comparing relative log_2_ fold change values of proteins for each isolate using 00–0949 as the reference when non-exclusive peptides were included in the Scaffold analysis. D. PCA comparing the first two components when non-exclusive peptides were included in the Scaffold analysis.

**Fig 4:**
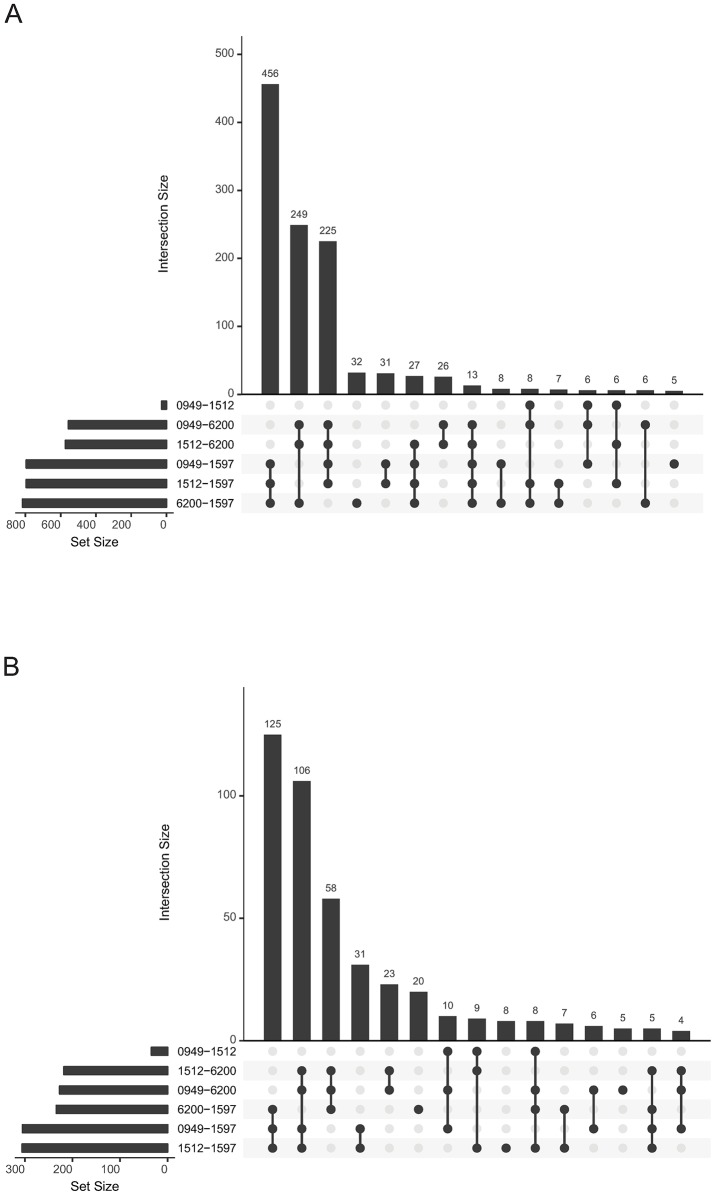
UpSet plots summarizing differential protein expression analysis for the four isolates. The horizontal bar graph at the bottom left of each figure shows the total number of proteins with differences in log_2_ fold change expression for each pair of isolates. Joined black circles to the right of these bar graphs indicate that the same differentially expressed proteins were common to the pairwise isolate comparisons shown at left. The vertical bar graph at the top quantitates the number of proteins with similar log_2_ fold change expression differences in the isolate comparisons. A. Results obtained using only exclusive peptides in the Scaffold pair-wise differential expression analyses. B. Results obtained when non-exclusive peptides were included in the Scaffold pair-wise differential expression analyses. Note the difference in scale between the two bar graphs.

Global proteome comparisons of the four isolates using comparative proteomic analysis with exclusive peptides demonstrates that the genetic variability is also seen among expressed proteins. Much less variability is detected within each isolate than between isolates, indicating good reproducibility of iTRAQ results (Fig 3A and 3C). Consistent with their genetic relatedness, isolates 00–0949 and 01–1512 exhibit fewer proteomic differences than the other two isolates, though they are distinguishable by a relatively small number of significantly differentially expressed proteins (Figs 3 and 4). All four isolates are clearly clustered and identifiable in PCA, with the HS:2 isolates 00–0949 and 01–1512 clustered more closely than the other two, more divergent, isolates (Fig 3B and 3D). We hypothesize that some of the results presented in Fig 3 may result from differential regulation in the different isolates. When conducting differential testing for each protein at the peptide abundance level, there were more differences between isolates in protein expression when only unique peptides were used for Scaffold analysis (Fig 4A) than when non-exclusive peptides were included (Fig 4B), suggesting that allelic differences between proteins captured by using only exclusive peptides in the analysis are responsible for some of the protein expression differences evident in the heat maps (see more detailed discussions below).

Gene ontology (GO) analysis utilizing statistically significant protein log_2_ fold change differences shows enrichment of specific biological processes among different sets of proteins from the different isolates ((S4 and S5 Spreadsheets)). A total of 2353 proteins were included in the GO enrichment analysis. More GO categories containing proteins with significantly increased or decreased expression levels are captured using data analyzed using exclusive peptides than with non-exclusive peptides. This is consistent with the hypothesis that some of the expression differences may be associated with specific alleles of a protein (analysis with exclusive peptides), while others are not dependent on allelic differences but may be due to differential regulation or differential presence/absence of the gene or protein (analysis using non-exclusive peptides). Comparisons of isolates 00–0949 and 01–1512 do not detect any significant differences in GO functions, consistent with the very close genetic identity between these isolates. Highly enriched GO categories in HS:2 isolates 00–0949 and 01–1512 compared with 00–6200 include those associated with biosynthetic processes involving alcohol and sugars, while comparisons of the HS:2 isolates to 00–1597 indicate the HS:2 isolates are most enriched with oxidoreductase activity and FMN binding (Table 3, analysis done using exclusive peptides). Compared with the HS:2 isolates, 00–6200 was enriched for expression of small molecule metabolic processes, (purine) nucleotide binding, and hydrolase activity. When compared with isolate 00–1597, 00–6200 was enriched in coenzyme/cofactor metabolic processes and cofactor binding. Isolate 00–1597, the most genomically divergent isolate of the four, is enriched for catalytic activity, nucleotide binding, and hydrolase activity compared with 00–0949, for metabolic and cellular processes compared with 01–1512, and with hydrolase activity compared with 00–6200 (Table 3). When non-exclusive peptides are included in the Scaffold analysis the results obtained are radically different (Table 4), meaning that no enrichment of GO categories is detected in isolates 00–0949 or 01–1512. When compared to the HS:2 isolates, isolate 00–6200 is enriched in with cellular and metabolic processes, but is depleted in these functions compared with 00–1597 (Table 4). Similar to the results obtained using exclusive peptides, isolate 00–1597 is enriched with catalytic activity and binding compared with all other isolates. As a whole the data suggest that the 00-0949/01-1512 isolate pair, isolate 00–6200, and isolate 00–1597 each had distinct patterns of regulation of protein expression. However, the difference in results obtained when only exclusive versus non-exclusive peptides are used for Scaffold analysis of relative protein expression indicates that each analytic strategy is measuring different properties of the protein content of the isolates. We decided to take an empirical approach to determining what those differences may be by comparing expression of proteins encoded by genes that were very closely related or identical in the four isolates.

**Table 3:** The most highly enriched and depleted GO categories in comparisons between isolates when analysis includes only exclusive peptides.

	Enrichment factor				
GO category enriched	00–0949 vs 00–6200	01–1512 vs 00–6200	00–0949 vs 00–1597	01–1512 vs 00–1597	00–6200 vs 00–1597
monosaccharide biosynthetic process	4.398	4.584			
alcohol biosynthetic process	3.998	4.167			
carbohydrate biosynthetic process	3.776	3.704			
cellular carbohydrate biosynthetic process	3.665	3.820			
alcohol metabolic process	3.529				
nucleotide-sugar metabolic process		4.775			
purine ribonucleotide binding	0.067	0.059			
purine nucleotide binding	0.066	0.059			
hydrolase activity	0.065	0.058			
nucleotide binding	0.057	0.050			
small molecule metabolic process	0.050	0.045			
oxidoreductase activity, acting on the CH-NH2 group of donors			5.252	5.264	
FMN binding			5.252	5.264	
acid-amino acid ligase activity			4.596	4.606	
transaminase activity			4.377	4.387	
sequence-specific DNA binding			4.377	4.387	
purine nucleotide binding			0.045		
hydrolase activity			0.044		
nucleotide binding			0.038		
binding			0.016		
catalytic activity			0.013		
biosynthetic process				0.023	
primary metabolic process				0.018	
cellular metabolic process				0.016	
cellular process				0.015	
metabolic process				0.015	
coenzyme metabolic process					2.309
cofactor binding					2.145
cofactor metabolic process					2.124
cellular nitrogen compound biosynthetic process					1.739
small molecule metabolic process					1.556
RNA binding					0.099
nucleoside-triphosphatase activity					0.099
pyrophosphatase activity					0.095
hydrolase activity, acting on acid anhydrides, in phosphorus-containing anhydrides					0.094
hydrolase activity, acting on acid anhydrides					0.094

Results were ordered by enrichment factor and filtered by statistical significance (Benjamini-Hochberg FDR ≤0.05).

Values > 1 indicate enrichment in the first isolate in each comparison; values < 1 indicate enrichment in the second isolate in each comparison (depletion in the first isolate in each comparison).

**Table 4:** Total enriched and depleted GO categories in comparisons between isolates when analysis includes non-exclusive peptides.

	Enrichment factor				
GO category enriched	00–0949 vs 00–6200	01–1512 vs 00–6200	00–0949 vs 00–1597	01–1512 vs 00–1597	00–6200 vs 00–1597
cellular process	0.190	0.181		0.035	0.123
primary metabolic process	0.157	0.149		0.043	0.153
cellular metabolic process	0.140	0.134		0.038	0.137
metabolic process	0.126	0.120		0.035	0.123
nitrogen compound metabolic process	0.103			0.056	0.134
catalytic activity			0.125	0.150	0.214
binding			0.117	0.187	0.267
nucleobase-containing compound metabolic process				0.091	
cellular macromolecule metabolic process				0.079	0.104
macromolecule metabolic process				0.075	0.099
cellular nitrogen compound metabolic process				0.060	0.143
cellular biosynthetic process				0.059	0.141
biosynthetic process				0.054	0.129
metabolic process				0.035	0.123

Results were ordered by enrichment factor and filtered by statistical significance (Benjamini-Hochberg FDR ≤0.05).

Values > 1 indicate enrichment in the first isolate in each comparison; values < 1 indicate enrichment in the second isolate in each comparison (depletion in the first isolate in each comparison).

Proteins produced by genes with 99% sequence identity or less are frequently distinguished into distinct alleles in the comparative experiments reported here when both exclusive and non-exclusive peptides are used for the comparative analysis (see sections below for examples). In most cases when exclusive peptides are used the log_2_ fold change differences for the alleles are comparable, suggesting the alleles are present at similar levels in their respective isolates. However, this phenomenon makes analysis of the comparative proteomics results much more complex. When non-exclusive peptides are used for iTRAQ analysis it becomes more difficult to reconcile log_2_ fold change differences with isolate identity.

#### CJIE4 prophage proteins are detected at different levels in different isolates

While the genomes of the closely related isolates 00–0949 and 01–1512 are generally syntenous, the location of the CJIE4 prophage is different (Fig 1). In isolates 00–0949, 00–6200, and 00–1597 CJIE4 is located between tRNA-Met and tRNA-Phe near flagellar glycosylation and capsule biosynthesis regions. In contrast, CJIE4 is located much closer to the origin of replication and in reverse orientation in isolate 01–1512, interrupting the gene encoding nucleoside hydrolase. CJIE4 is therefore mobile, at least within the *C*. *jejuni* genome, and is not necessarily associated with tRNA genes. A comparison of CJIE4 prophages in the 4 isolates, along with the proteins expressed from these prophages, has been discussed previously [33], though differences in protein detection levels was not included in that discussion. The most striking difference within CJIE4 is the presence of an indel, with one variant comprised of 5 genes (encoding the Cro/CI family transcriptional regulator, hypothetical protein PJ17_06640, 3′-5′ exonuclease, hypothetical protein PJ17_06650, NTPase KAP) present in isolates 00–0949 and 00–1597 and the alternate indel of 4 genes (encoding hypothetical protein PJ18_6440, peptidase S24, endonuclease, and hypothetical protein PJ18_06455) present in isolates 00–6200 and 01–1512 [33]. All proteins except hypothetical protein PJ18_06440 are detected at significantly higher log_2_ fold change in the appropriate isolates, consistent with the presence or absence of the genes (S2 Table). Several proteins not present in either large indel variant and closely associated with prophage function are detected at higher log_2_ fold change values only in isolate 00–0949, suggesting that they may be differentially regulated in this isolate. These include Emm, hypothetical protein PJ16_07060 (CJE1444 homolog), hypothetical protein PJ17_06675 (CJE1447 homolog), both alleles of the CJE1452 homolog, capsid protein PJ17_06720 (CJE1458 homolog), DNA repair protein PJ17_06750 (CJE1465 homolog), and at least one variant of the CJE1466 homolog (S2 Table). CJIE4 integrase is detected at higher levels in isolate 01–1512 and the majority of the remaining proteins are detected at similar levels in all isolates except those encoded by genes encoding a hypothetical protein (PJ18_06560) and RelE (PJ18_06560), which are found only in isolate 00–6200. Results obtained from analyses with both exclusive and non-exclusive peptides were similar except for RM1221 homologs of CJE1452, CJE1466, CJE1477, and RelE. The DNA sequence-independent strain variation in detection of numerous CJIE4 prophage proteins suggests the potential for independent regulation of expression of these proteins in different isolates and further indicates that comparative proteomic experiments can detect differential regulation between isolates. It would be interesting to determine whether the factors associated with this putative regulation are encoded within the prophage itself or in the chromosome outside CJIE4.

#### CJIE1 and a CJIE1 variant prophage have genetic differences and proteins from both are expressed

CJIE1 prophages are present only in the two HS:2 isolates 00–0949 and 01–1512 (Figs 1 and 2). Both isolates harbor two prophages, CJIE1 and a variant closely related to CJIE1. There are 27 gene/protein homologs common to both prophage variants, 33 found only in CJIE1, and 27 found only in the CJIE1 prophage variant (S3 Table). The extensive amount of genetic material common to both prophage variants creates long sequence repeats of 7141 bp, 2626 bp, 1567 bp, and 1387 bp that may be capable of mediating chromosomal rearrangements by homologous recombination, as well as two shorter repeats of <400 bp. Large genomic rearrangements subsequent to lytic bacteriophage predation have previously been attributed to recombination between genes associated with CJIE1 and CJIE1 variant bacteriophages [31]. A hypothetical protein homologous to CJE0229 of *C*. *jejuni* strain RM1221 is present in the CJIE4 prophage as well as in both CJIE1 and CJIE1 variant prophages. CJIE1 interrupts a gene encoding histidine kinase in both isolates, a different location than in either RM1221 [49] or the Walkerton outbreak clone represented by isolate 00–2425 [50]. CJIE1 has previously been detected at different locations in *C*. *jejuni* genomes, suggesting it may integrate randomly [9]. The CJIE1 variant prophage interrupts an arsenic transporter in both isolates (PJ16_06170 in 00–0949, PJ19_06435 in 01–1512). The tail tape measure proteins, bacteriocin (phage Mu transposition protein B) and integrase (phage Mu transposition protein A) are very different in the two prophage variants at 27%, 33%, and 30% identity, respectively, with incomplete coverage of each of the proteins compared. Despite long regions of identity, we speculate that these prophages may have different biological properties in addition to inactivation of the genes in which they are inserted.

As expected, CJIE1 and CJIE1 prophage variant proteins are not detected (assessed as very negative log_2_ fold change values) in isolates 00–6200 and 00–1597, which do not carry these prophages (S3 Table). In three cases there was no detection of proteins when analysis of 4-plex iTRAQ experiments was done with exclusive peptides, while inclusion of non-exclusive peptides resulted in interpretable data consistent with the known presence and absence of the prophages in the four isolates (S3 Table). The observation that 8/16 CJIE1 proteins and 1/3 CJIE1 prophage variant proteins were detected in fewer than 3 replicate experiments suggests they may have been expressed at levels at or near the limit of detection or that they were inconsistently induced. Interestingly, two proteins present in an indel associated with a DNA-binding protein and deoxyribonuclease (hypothetical proteins PJ16_03565 and PJ16_03570) were detected in all six replicate experiments, consistent with the high level of expression previously observed for these proteins [22].

#### Proteins encoded by genes within the Type VI secretion system genomic island (T6SS island) unique to isolate 00–1597 are detected in that isolate

Isolate 00–1597 harbors a large indel of approximately 60-kb containing 52 genes unique to the isolate (Fig 1) inserted between tRNA-Arg (PJ17_04940) and a gene encoding a membrane protein (PJ17_05205), which are adjacent in isolates 00–0949, 01–1512, and 00–0949. Within this region there are 13 genes in an ~18 kb region encoding components of a Type VI secretion system (T6SS gene cluster, S4 Fig). The organization of this Type VI gene cluster is identical to that *of C*. *jejuni* strain 108 (GenBank Accession No. JX36460) and also appears to be inserted into CJIE3 [51]. The T6SS island containing the type VI secretion system (T6SS) is closely related to a similar region in strain NCTC11351 (GenBank Accession No. LN831025). Both isolate 00–1597 and NCTC11351 harbour the complete T6SS gene cluster, while in RM1221 it appears that much of this gene cluster may have been deleted (see S4 Fig) [51,52]. Consistent with this hypothesis, the second intact gene/protein belonging to the Type VI secretion cluster (*tssM*, *vasK* homolog) in isolate 00–1597 has been annotated as a Type VI secretion system pseudogene, CJE_RS05700, in the RM1221 reference genome (GenbankAccession No. NC_003912.7). Several of the genes/proteins in the T6SS genomic island appear to be homologous to plasmid genes, and blastn identity was also found with *C*. *jejuni* strain OD627 plasmid pCJDM67 L (GenBank Accession No. CP014745) and *C*. *jejuni* strain WP2202 plasmid pCJDM202 (GenBank Accession No. CP014745). This is consistent with the T6SS island and CJIE3 existing as both a plasmid and a chromosomal integrated element [49] as well as the detection of the T6SS gene cluster in megaplasmids [53]. The T6SS effector Hcp/major exported protein of isolate 00–1597 shows 100% identity with many *C*. *jejuni* HCP proteins, including the HCP from *C*. *jejuni* strain 108 described by Bleumynk-Pluym and colleagues [51].

Results from comparative proteomic experiments indicated that T6SS island proteins are expressed in isolate 00–1597 (S4 Table) and are not detected (present at much lower log_2_ fold change values) in 00–0949, 01–1512, and 00–6200, consistent with the absence of the T6SS island in the latter 3 isolates. The proteins detected in bacterial pellets include homologs of RM1221 CJIE3 proteins CJE1110, CJE1138, CJE1139, CJE1141, CJE1150, and CJE1153, as well as homologs of TagH (VasC), TssM (VasK), TssD (major exported protein HCP), TssK (VasE), TssJ (VasD), TssA (VasJ), TssB (VipA), and TssC (VipB) using the comparative terminology established by Bleumink-Plym and colleagues [51]. These results confirm the previous observation that expression of T6SS cluster genes is constitutive under standard bacterial growth conditions [51]. Differences in log_2_ fold change values for the T6SS island proteins are highly significant for proteins detected in 5 or more replicate experiments, but have lower or no statistical significance for proteins detected in 2 or fewer replicate experiments despite the fact that the genes encoding these proteins are present only in isolate 00–1597 (S4 Table). Six non-T6SS proteins encoded by genes within the larger T6SS island were also detected. These were all annotated as hypothetical proteins, so it is not possible to infer their contribution to the biology of the organism. Results are consistent between analyses with exclusive or non-exclusive peptides, except for hypothetical proteins PJ17_05075 and PJ17_05075, which are detected only when non-exclusive peptides are included. The T6SS gene cluster has been associated in different bacterial species with virulence within the host and killing of neighboring bacteria [54,55], and in *C*. *jejuni* with contact-dependent hemolysis in a capsule-deficient bacterial phenotype [51]. Co-culture of *C*. *jejuni* with HCT-8 epithelial cells results in loss of capsular polysaccharide expression due to downregulation of the genes in the capsule biosynthesis locus, but also results in reduced invasion [56]. While Siddiqui et al. [57] did not detect the T6SS gene cluster or expression of T6SS genes in isolates from humans infected with *C*. *jejuni*, others have detected the T6SS cluster in a relatively high proportion of human patients with *Campylobacter* bloody diarrhea or bacteremia [51,52]. Therefore, the T6SS may be responsible for survival of *C*. *jejuni* in particular environments as well as contributing to virulence of the organism.

#### The *C*. *jejuni* invertible region shows heterogeneity in size and gene content

There is an approximately 88 to 96-kb region of *C*. *jejuni* genomes flanked by genes encoding chemotaxis proteins, also known as transducer-like proteins (Tlps), in approximately the same location in all 4 isolates (Fig 1). This region is inverted in isolate 00–6200 relative to the other 3 isolates, a phenomenon previously seen in comparisons between strain NCTC11168 = ATCC 700819 and 4 clonal *C*. *jejuni* from the Walkerton outbreak [41]. The Tlps flanking the invertible element are the same in the more closely related isolates 00–0949 and 01–1512, but are divergent in the other two isolates. The gene content of the invertible region is similar in all 4 isolates and there is overall synteny; however, differences in the size of the invertible region indicate that there are some differences in gene content. In isolate 00–1597 the invertible region harbours an indel consisting of 5 genes encoding the following proteins: transposase (PJ17_01170); hypothetical protein (PJ17_01175), hydrogenase expression protein HypA (PJ17_01180); pyrrolidone carboxylate peptidase (PJ17_01185); hypothetical protein (PJ17_01190). Therefore, though the invertible region is mostly syntenous in different isolates, it would also appear to be a target for recombination resulting from horizontal gene transfer. Only 2/5 proteins from the 00–1597 indel (hydrogenase expression protein HypA and hypothetical protein PJ17_01190) were detected in comparative proteomics experiments. The hypothetical protein was detected at much higher levels in isolate 00–1597. However, hydrogenase expression protein HypA was detected at similar levels in isolates 00–0949, 01–1512, and 00–1597, a finding inconsistent with evidence that the gene encoding this protein was present only in isolate 00–1597. Inspection of the protein in Scaffold indicated several peptides were shared in the 00–1597, 00–0949, and 01–1512 isolates but not in the 00–6200 isolate. Searches of the GenBank files of the four isolates found the peptides only in isolate 00–1597, indicating a rare error in the proteomics results. Finally, the gene encoding membrane protein PJ17_09055 has high DNA sequence identity with disrupted genes in isolates 00–0949 and 01–1512 and low DNA sequence identity with the homolog in isolate 00–6200; the protein is correctly detected only in 00–1597 with use of either exclusive or non-exclusive peptides in the analysis.

Variants of many of the genes within the invertible region were present in different isolates, and comparative proteomics experiments distinguished almost all of the resulting protein variants (S2 and S3 Spreadsheets). The size of the region and the existence of alleles or variants of so many different proteins make a detailed description of differential protein detection in this region too complex for discussion here. Though the entire region was inverted in isolate 00–6200 relative to the other two isolates there were no consistent differences in protein expression that were orientation-specific; in other words, that were consistently higher nearer the chromosomal origin of replication in the inverted versus non-inverted regions of the different isolates (S2 and S3 Spreadsheets).

Additional differences between isolates are in previously characterized hypervariable regions, especially the capsular polysaccharide biosynthesis gene cluster, the flagellar glycosylation gene cluster and, to a lesser extent, the lipo-oligosaccharide (LOS) biosynthesis locus (Figs 1 and 2).

#### CPB gene clusters show heterogeneity in genes and protein expression

Capsular polylsaccharides are the major antigens in the Penner heat-stable serotyping scheme [58]. Genes associated with capsular polysaccharide export (*kpsS*, *C*, *D*, *E*, *T*, and *M*) are common to all CPB gene clusters within *Campylobacter* genomes [59]. None of these six genes is completely identical in all four isolates characterized here; nucleotide sequence identities range from 85–100%, depending on the isolates compared. Except for *kpsE*, which was identical in all isolates except 00–6200, the most divergent genes were harboured by isolate 00–1597 (S5–S7 Tables). The capsule biosynthesis protein (*kpsM* gene) is not detected in comparative proteomic experiments. Variants of the other capsular polysaccharide conserved export proteins are detected at similar levels. The greatest divergence in sequence identity identified among the four isolates for the capsular polysaccharide export genes is in *kpsC* and *kpsS* of HS:9 isolate 00–1597 (85% and 88%, respectively. Capsule biosynthesis protein (KpsC) is detected in all 4 strains, though the alleles associated with HS:2 strains 00–0949 and 01–1512, the HS:4 strain 00–6200, and the HS:9 strain 00–1597 are differentiated from each other (compare S5–S7 Tables). Both the HS:9 and HS:2 KpsC alleles are most highly expressed in their homologous isolates when exclusive and non-exclusive peptides are used in the analysis, while the 00–6200 KpsC and KpsE alleles appear to be expressed at higher log_2_ fold change values only when exclusive peptides were used for analysis (S6 Table), suggesting comparative proteomics experiments are capable of differentiating the 00–6200 alleles from those in other isolates. Despite a 99% nucleotide sequence identity, the sugar ABC transporter substrate-binding protein (*kpsD* gene) and arabinose 5-phosphate isomerase (*kpsF* gene) variants in isolate 00–1597 are differentiated from those in the other three isolates and allele-specific expression is detected. KpsT (ABC transporter ATP-binding protein) is detected at similar log_2_ fold change levels in all four isolates. There is heterogeneity in this set of genes/proteins common to all *C*. *jejuni* capsule biosynthesis gene clusters, and comparative proteomics is capable of differentiating proteins encoded by genes differing by only 1% nucleotide identity. This complicates analysis and interpretation of the results.

The HS:9 CPB gene cluster in isolate 00–1597 is 99% identical at the DNA level to the one reported previously (Genbank Accession No. KT868844 [60]; see S5 Fig). All loci unique to the 00–1597 HS:9 CPB except UDP-galactopyranose mutase (80% nucleotide identity with its homolog in the HS:2 CPB) have <50% DNA sequence identity with either the HS:2 or HS:4 CPBs. All unique proteins encoded within the HS:9 CPB gene cluster are expressed and detected only in isolate 00–1597 (S6 Table) when either exclusive or non-exclusive peptides are used for the analysis.

The 00–6200 HS:4 CPB gene cluster has 10,311/10,541 (98%) nucleotide identity with 22/10,541(0%) gaps when compared with strain ATCC 43432, which is a reference strain for the HS:4 capsule locus [59,60] along with HS:4 complex strain CG8486 [61]. However, 00–6200 CPB gene cluster has greater identity with the cluster from YH001 at 37,085/37,363 nucleotide identity (99%) and 28/37,363 gaps (0%) (S6 Fig). Both the 00–6200 and YH001 HS:4 CPB gene clusters have an insertion of 5 genes (in order: alpha-2,3-sialyltransferase, glycosyltransferase family 2, capsular biosynthesis protein, uridine kinase, and hypothetical protein PJ18_07260) compared with the ATCC 42432 CPB gene cluster (see loci C–G, S6 Fig), and differ in the 3′ half of a gene encoding a MeOPN transferase (00–6200 locus PJ18_07330; see locus 16, S6 Fig). Differences in HS:4 CPB loci have been demonstrated previously, and at least some of the predicted proteins in the 00–6200 insertion are similar to strain 12,which has a Group A CPB cluster [15]. The first two of the five additional genes are expressed under laboratory growth conditions (S6 Table). HS:2 and HS:4 gene clusters harbour homologs with high levels of nucleotide identity as well as unique genes and proteins (S6 and S7 Tables). Several proteins (PJ18_07260 to PJ18_07285; PJ18_07315 to PJ18_07335), including the insertion, are unique to HS:4 isolate 00–6200 in this study and two of these are detected at higher log_2_ fold change values in isolate 00–6200 when exclusive or non-exclusive peptides are used for analysis (S6 Table). Several protein alleles encoded by genes with 91% identity or greater to genes in the two HS:2 isolates are detected at much higher log_2_ fold change values in isolate 00–6200 when either unique or non-exclusive peptides are used in the analysis (S6 Table). In contrast, proteins at loci PJ18_07235, PJ18_07300, and PJ18_07305 are encoded by genes with >96% identity to alleles in the HS:2 CPB locus. These proteins were detected at much higher log_2_ fold change values in isolate 00–6200 only when exclusive peptides are used for analysis, indicating that the putative expression differences were likely an artefact resulting from the allelic differences since only the tryptic peptides responsible for the alleles would be used to score presence and absence of the protein. The two methyltransferases will be discussed below.

The HS:2 CPB are identical in isolates 00–0949 and 01–1512 except for the gene encoding UDP pyrophosphate phosphatase (S7 Table). In addition to the *kpsS*, *C*, *F*, *D*, *E*, *T*, and *M* genes discussed earlier, nine genes are also shared at greater than 90% identity with the CPB cluster in isolate 00–6200 while the remaining seventeen genes are unique. Almost all unique genes express proteins at much higher log_2_ fold change values when either exclusive or non-exclusive peptides are used for analysis (S6 Table). Genes encoding sugar transferase in isolates 00–0949 (PJ16_07840) and 01–1512 (PJ19_07835) share 100% nucleotide identity; expression is controlled by a homopolymeric tract. The protein is detected in isolate 00–0949 at a much higher log_2_ fold change value than in isolate 01–1512 whether non-exclusive peptides are included or not. It would appear that some factor results in differential expression (differential regulation) of the protein in the two strains. The next gene in the cluster also encodes a sugar transferase (00–0949, PJ16_07845; 01–1512, PJ19_07840), has 100% identity in both isolates, and expression is controlled by a homopolymeric tract. However, the protein is detected at equivalent levels (log_2_ fold change values) in both HS:2 isolates. Several genes with between 91–99% identity between the HS:2 and HS:4 CPB gene clusters produce protein variants distinguishable in comparative proteomic experiments (S6 and S7 Tables). Each variant is detected at much higher log_2_ fold change values in the isolate in which it is present when non-exclusive peptides are included in the analysis. Two methyltransferases (PJ16_07830/ PJ18_07250 and PJ16_07835/PJ18_07255) are present in both the HS:2 and HS:4 CPB gene clusters at 99% and 98% identity, respectively. The first methyltransferase is detected in comparative proteomics experiments at much higher log_2_ fold change values isolate 01–1512 than in HS:2 isolate 00–0949 or HS:4 isolate 00–6200 whether non-exclusive peptides are included in the analysis or not (S6 and S7 Tables). The second methyltransferase is controlled by a homopolymeric tract in all three strains and is detected at higher log_2_ fold change levels in isolates 00–0949 and 00–6200 than in HS:2 isolate 01–1512 when exclusive or non-exclusive peptides are included in the analysis (S2 Spreadsheet, S6 and S7 Tables). This is consistent with regulatory differences between 01–1512 and the other two isolates.

Analysis of comparative proteomics data from comparative protein expression experiments with exclusive and non-exclusive peptides frequently appears to provide different results, such that it may be necessary to analyze results with both for a full understanding of factors affecting protein expression.

#### Transducer-like chemotaxis protein variants are detected only when non-exclusive peptides are not included in analysis

Chemotaxis proteins encoded by chromosomal genes are integral to virulence and niche adaptation in *C*. *jejuni* [62,63]. The genes encoding group A chemoreceptor proteins, also known as transducer-like proteins (Tlps), are very divergent in their N-terminal half, allowing classification of different Tlp types, and have high identity in their 3′ half, creating extensive regions of identity among the different *tlp* genes (chromosomal repeats) [64] that appear to mediate large chromosomal inversions [41]. Genes encoding group A Tlps in the 4 isolates were identified and Tlp proteins were detected in comparative proteomics experiments. Genes encoding Tlp1 and Tlp3 are present in all 4 isolates (S8 Table); lower nucleotide identity indicates different N-termini and therefore different Tlps. Two copies of *tlp3* genes were detected in the 00–6200 genome (not shown), which would result in an even longer chromosomal repeat. The Tlp proteins encoded by these two genes (PJ18_00730 and PJ18_07900) share 99% identity, but only one of the alleles was detected in comparative proteomic experiments (S8 Table). Analysis using exclusive peptides indicates that the log_2_ fold change values were very similar for Tlp3 in isolates 00–0949, 01–1512, and 00–6200 despite higher gene dosage in 00–6200. The 00–6200 Tlp3 allele is not detected in isolate 00–1597 (S8 Table). Results of analysis including non-exclusive peptides suggest similar detection levels for Tlp3 proteins from all four isolates, as well as detecting the Tlp3 allele in isolate 00–1597. Examination of the “Proteins” field in Scaffold for Tlp3 in isolate 00–1597 determined that 2 unique tryptic peptides were detected with high probability compared with 30 non-exclusive peptides, but that only one of the exclusive peptides was used to derive fold change values. Since the number of peptides required for reporting of results was set at 2, no result was obtained for isolate 00–1597 when exclusive peptides were used for analysis. Similar explanations may account for other situations in which proteins are not detected using unique peptides but are detected when non-exclusive peptides are used.

Tlp1 proteins share at least 99% identity among the 4 isolates and are 100% identical in 00–0949 and 01–1512. The variation evident from comparative proteomics experiments was not captured by LS-BSR, perhaps because we used nucleotide fasta files to implement the analysis and that would require ORF prediction, which may be different in LS-BSR analysis than the annotated sequence in NCBI. The 00–0949 and 01–1512 Tlp1 allele is expressed at much higher log_2_ fold change values than alleles in 00–6200 and 00–1597 when either exclusive or non-exclusive peptides are used in the analysis. However, for the 00–6200 allele (PJ18_07650) the use of non-exclusive peptides for analysis results in roughly similar detection levels for Tlp1 in all isolates. The *tlp4* gene is present only in isolate 01–1512 but differential expression of the protein is higher in this isolate only when unique peptides are used for analysis (S8 Table). This is likely an artefact that can be attributed to the large region of identity (repeat region) characteristic of this class of Tlps, which could tend to homogenize detection levels. Tlp11 was found previously, though misclassified as a novel Tlp [41], and the *tlp11* gene is unique to 00–6200 among the four isolates in the current analysis. The Tlp11 protein has a much higher log_2_ fold change value in this isolate when exclusive peptides are used in the analysis but has similar log_2_ fold change value in all isolates when non-exclusive peptides are included. Recently published work describes the identification of two novel Tlps, Tlp12 and Tlp13, as well as the distribution of Tlps within the *C*. *jejuni* population [65]. Other novel Tlps have been characterized in the 4 isolates analyzed for this work. The *tlp12* and *tlp13* genes are unique to isolate 00–01597, and the log_2_ fold change value for these proteins are significantly higher in 00–1597 when analysis is done both with and without non-exclusive peptides (S8 Table). We have designated Tlp14 as a novel Tlp encoded by the *tlp14* gene on the basis of a lack of identity with other Tlps in the N-terminal substrate-binding region. Tlp14 is present in isolates 01–1512, 00–0949, and 00–01597. Differential detection was noted between the alleles in the HS:2 isolates versus the 00–1597 allele whether or not exclusive or non-exclusive peptides were used for analysis (S8 Table). In aggregate the data indicate that the structure of Tlp proteins affects the results from comparative proteomics analysis. The use of exclusive and non-exclusive peptides for the analysis appears to provide different results by assessing either the N-terminal half (exclusive peptides) or C-terminal half (non-exclusive peptides) or possibly both (Tlp14 in isolate 00–6200; S8 Table).

#### Iron acquisition proteins and oxidative stress proteins are detected at higher levels in isolate 00–1597

Chromosomally encoded iron acquisition and oxidative stress alleles exhibit between 97–100% nucleotide identity with the exception of the gene encoding the 11 kDa thioredoxin; the 00–6200 allele has 81% nucleotide identity with the other common allele (S9 Table). Alleles of the ferrous iron transporter A, as well as the 21 kDa thioredoxin protein, are expressed at roughly similar levels. Several iron acquisition proteins are generally detected at much higher log_2_ fold change levels in isolate 00–1597 than in the other three isolates (S9 Table). One of the two biopolymer transporter ExbD proteins (PJ16_08550) exhibits elevated log_2_ fold change values in isolate 00–1597 when non-exclusive peptides are used for the analysis; this protein would appear to be co-regulated with the other iron acquisition proteins listed above. However the biopolymer transporter ExbD allele associated with isolates 00–1597 and 00–6200 (PJ17_00560 in S9 Table) exhibits differential protein expression only when exclusive peptides are included in the analysis. The two alleles have a different content of exclusive peptides but do not appear to be co-regulated with some of the other iron acquisition proteins in isolate 00–1597. Iron permease protein alleles are similarly differentiated, with great differences in log_2_ fold change values between the allele present in isolate 00–6200 (PJ18_08390) compared with the other three isolates apparent only when exclusive peptides are included in the analysis. When non-exclusive peptides are included, all iron permease alleles are detected at higher levels in isolate 00–1597. Together these results suggest that the high level of identity among the functionally similar proteins generates artefacts in the expression data when only unique peptides are included in the analysis, likely due to the very small number of tryptic peptides responsible for differentiation. Proteins that are not detected at all when only unique peptides are used include the biopolymer transporter ExbD (PJ16_08850; PJ17_08495) and the hemin transporter associated with isolates 00–6200, 00–0949, and 01–1512 (PJ18_08179; PJ16_8770; PJ19_9760) (S9 Table). In other cases, allelic variants of proteins are distinguished and return different log_2_ fold change values for the same protein. The observation that many iron acquisition proteins are detected at much higher levels in isolate 00–1597 than in the other 3 isolates suggests that isolate 00–1597 regulates expression of iron acquisition proteins differently than the other three isolates. Proteins associated with oxidative stress are regulated in concert with iron acquisition proteins [66]. Our results indicate that catalase and peroxidase are both detected at much higher log_2_ fold change values in isolate 00–1597 (S9 Table). Peroxiredoxin variants have different log_2_ fold change values when exclusive peptides are used for comparative proteomics analysis and similar log_2_ fold change values when non-exclusive peptides are used, suggesting the apparent differences result from the variation in protein sequence and not differences in protein expression. Only one of five thioredoxin proteins harbored by the isolates, the 18 kDa thioredoxin (PJ16_09020), exhibits higher log_2_ fold change values in isolate 00–1597 when either exclusive and non-exclusive peptides are used for analysis, suggesting this is the only thioredoxin that is co-regulated with iron-acquisition proteins.

#### Additional proteins exhibit differential detection in the four isolates suggestive of differences in regulation

A number of other chromosomally encoded proteins are detected at higher levels in one or two isolates compared with the others (S10 Table). Several are detected at higher log_2_ fold change values in isolate 00–6200 whether unique or non-exclusive peptides are used for data analysis, including an ABC transporter substrate binding protein, altronate hydrolase, aspartate-ammonia-lyase, dihydropicolinate synthase, a nitroreductase, a short-chain dehydrogenase, and trimethylene N-oxide reductase catalyltic subunit. A 30 kDa beta-lactamase and both the 93 and 120 kDa lipoproteins are expressed at higher levels in isolate 01–1512 when non-exclusive peptides are used, but the use of exclusive peptides allows differentiation of the HS:2/HS:4 allele from that of isolate 00–1597. The 23 kDa beta-lactamase is expressed at similar levels in all isolates and metallo-beta-lactamase is expressed at lower levels in isolate 00–6200 (S2 and S3 Spreadsheets). A membrane protein is expressed at much lower levels in isolate 00–0949 than in the other three isolates. Ferritin is more highly expressed in isolates 00–6200 and 00–1597 (S2 Spreadsheet and S10 Table). The differences do not appear to result solely from the absence of genes encoding the proteins of interest or large differences in gene identity, and overall suggest they may result from differential regulation. In most cases the difference in protein detection levels is seen regardless of whether non-exclusive peptides are included in the analysis. However in some cases (flagellar basal body rod protein FlgC, 120 kDa lipoprotein, molybdenum cofactor biosynthesis proteins MoaA and MoaE, 27 kDa oxidoreductase) there is a difference in results when non-exclusive peptides are included in the analysis versus when they are not.

## Conclusions

The study, which dealt with the presence or absence of genes and proteins and not SNPs or epigenomic phenomena, determined that the four isolates exhibited genetic differences among them consistent with their serotypes and MLST types. Though the most striking differences among isolates were in the hypervariable regions, prophage content, and Type VI secretion island, there were also differences in a subset of proteins associated with iron acquisition as well as some proteins associated with metabolism. GO category enrichment detects differences among isolates but does not capture the fine specificity of differences evident in a more focused analysis. The variability in size of the *C*. *jejuni* invertible chromosomal region highlights the capacity for acquisition or loss of genes in a relatively short part of the genome.

The ability of comparative proteomics experiments to distinguish even closely related proteins in different isolates greatly increases the complexity of the analysis on a genome/proteome-wide basis. Inclusion of non-exclusive peptides allows quantitation of relative protein amounts while variant protein alleles or isoforms are differentiated by using exclusive peptides. The nature of the protein must also be considered, in that proteins containing both conserved and unique domains may require the use of both exclusive and non-exclusive peptides in comparative proteomic analysis to understand expression data. Proteins encoded by unique genes are detected and quantitated equally well when both exclusive and non-exclusive peptides are used. Proteins encoded by genes with complete identity frequently had no data associated with them in comparative proteomic experiments when exclusive peptides were used. These proteins were detected when non-exclusive peptides were used.

The availability of closed, complete genomes greatly facilitated interpretation of the iTRAQ data. Analysis of comparative proteomics data obtained using exclusive and non-exclusive peptides frequently appears to provide different results, such that it may be necessary to analyze results with both for a full understanding of factors affecting protein expression. Protein expression differences between the four isolates were consistent with the genetic differences of the isolates. Comparative proteomics experiments allow the quantification of protein expression differences that may result from differential regulation in different isolates. This type of comparative quantitation will also be valuable for identification of proteins that are co-regulated.

## Supporting information

S1 Spreadsheet Statistical testing of isolate pairs. (XLSX)

S2 Spreadsheet Samples reports (Scaffold) containing iTRAQ data from experiments using unique peptides. (XLSX)

S3 Spreadsheet Samples reports (Scaffold) containing iTRAQ data from experiments using non-exclusive peptides. (XLSX)

S4 Spreadsheet GO enrichment analysis comparing pairs of isolates analyzed using unique peptides only. (XLSX)

S5 Spreadsheet GO enrichment analysis comparing pairs of isolates analyzed with inclusion of non-exclusive peptides. (XLSX)

S1 Fig Core genome of the four isolates. The images were obtained using GView Server, combined, and further annotated using Adobe Illustrator.
(PDF)

S2 Fig Accessory genome of the four isolates. The images were obtained using GView Server, combined, and further annotated using Adobe Illustrator.
(PDF)

S3 Fig Unique genome of the four isolates. The images were obtained using GView Server, combined, and further annotated using Adobe Illustrator.
(PDF)

S4 Fig Type VI secretion system gene cluster and island in isolate 00–1597 compared with isolate NCTC 11351 and CJIE3 of isolate RM1221. The images were obtained using GView Server, combined, and further annotated using Adobe Illustrator.
(PDF)

S5 Fig HS:9 capsular polysaccharide biosynthesis gene cluster of isolates 00–1597 and ATCC43437 (Accession No. KT868844). The images were obtained using GView Server, combined, and further annotated using Adobe Illustrator.
(PDF)

S6 Fig HS:4 capsular polysaccharide biosynthesis gene cluster of isolates 006200, YH001, and ATCC43432. The images were obtained using GView Server, combined, and further annotated using Adobe Illustrator.
(PDF)

S1 Table Homopolymeric (poly G) tracts in genomes of the four *C*. *jejuni* isolates. (DOCX)

S2 Table Prophage CJIE4 protein detection in the four *C*. *jejuni* isolates using comparative iTRAQ proteomic analysis. (DOCX)

S3 Table CJIE1 and CJIE1 variant prophage proteins and detection of proteins in 4-plex iTRAQ comparative proteomics experiments. (DOCX)

S4 Table Detection of proteins in the 00–1597 Type VI secretion island using comparative 4-plex iTRAQ proteomic analysis. (DOCX)

S5 Table Detection of proteins in the 00–1597 HS:9 CPB cluster using comparative 4-plex iTRAQ proteomic analysis. (DOCX)

S6 Table Detection of proteins in the 00–6200 HS:4 CPB cluster using comparative 4-plex iTRAQ proteomic analysis. (DOCX)

S7 Table Detection of proteins in the 00–0949 and 01–1512 HS:2 CPB cluster using comparative 4-plex iTRAQ proteomic analysis. (DOCX)

S8 Table Detection of transducer-like proteins (Tlps) using comparative 4-plex iTRAQ proteomic analysis. (DOCX)

S9 Table Detection of selected iron acquisition and oxidative stress proteins in the four *C*. *jejuni* isolates using comparative iTRAQ proteomic analysis. (DOCX)

S10 Table Selected proteins exhibiting differential detection in the four *C*. *jejuni* isolates using comparative iTRAQ proteomic analysis. (DOCX)
