# Supplementary figures and images for: Comparison of genomes and proteomes of four whole genome-sequenced Campylobacter jejuni from different phylogenetic backgrounds
Source: PLoS One. 2018 Jan 2;13(1):e0190836. doi: 10.1371/journal.pone.0190836 (PMC5749857; doi:10.1371/journal.pone.0190836)

Figure S1

00-0949 Core genome

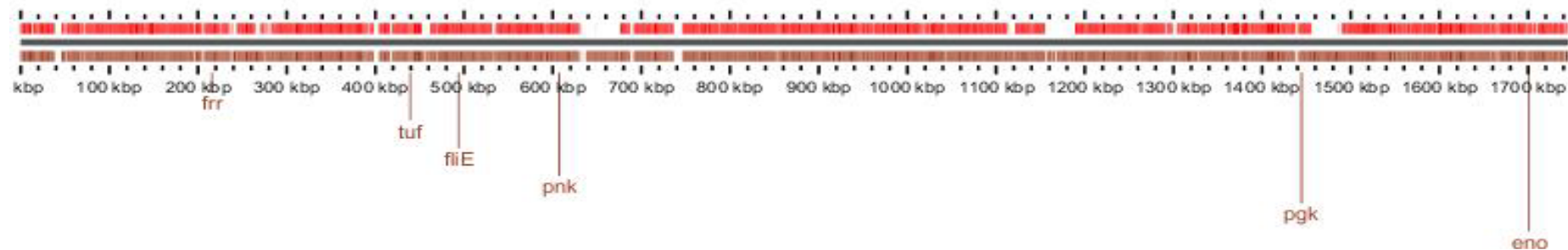

01-1512 Core genome

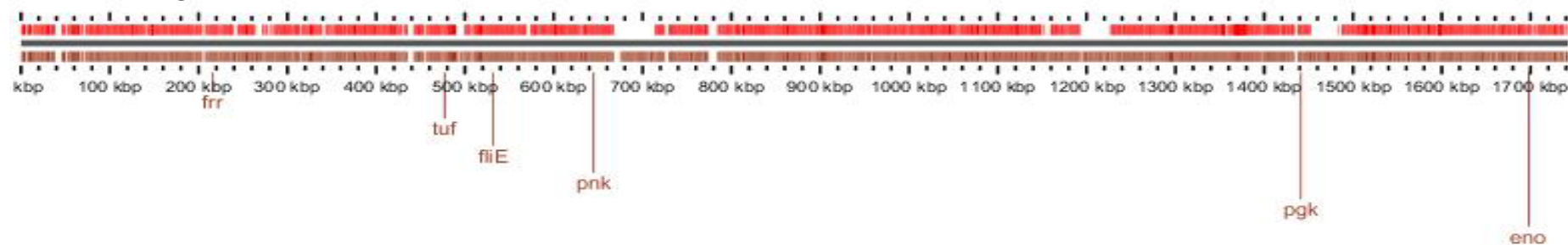

00-6200 Core genome

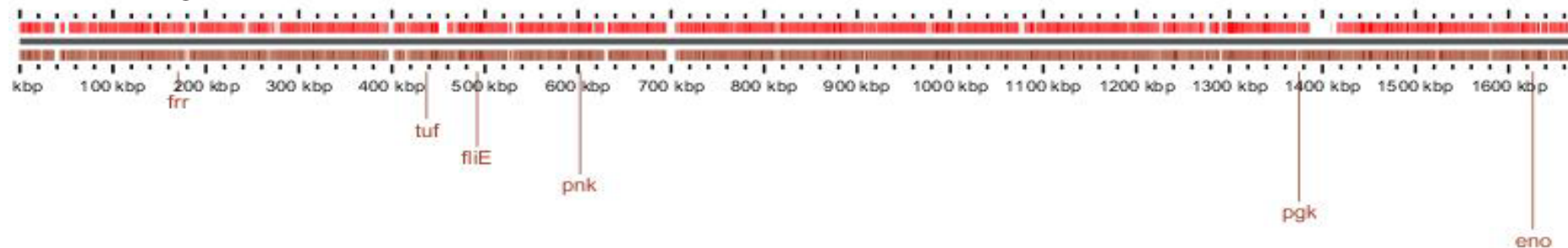

00-1597 Core genome

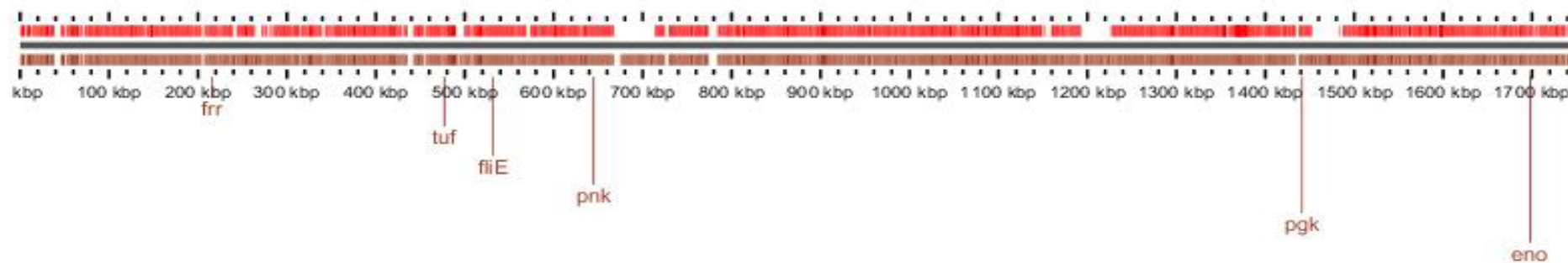

Supplement: S1 Fig — The images were obtained using GView Server, combined, and further annotated using Adobe Illustrator. (PDF) [file pone.0190836.s006.pdf]

Figure S2

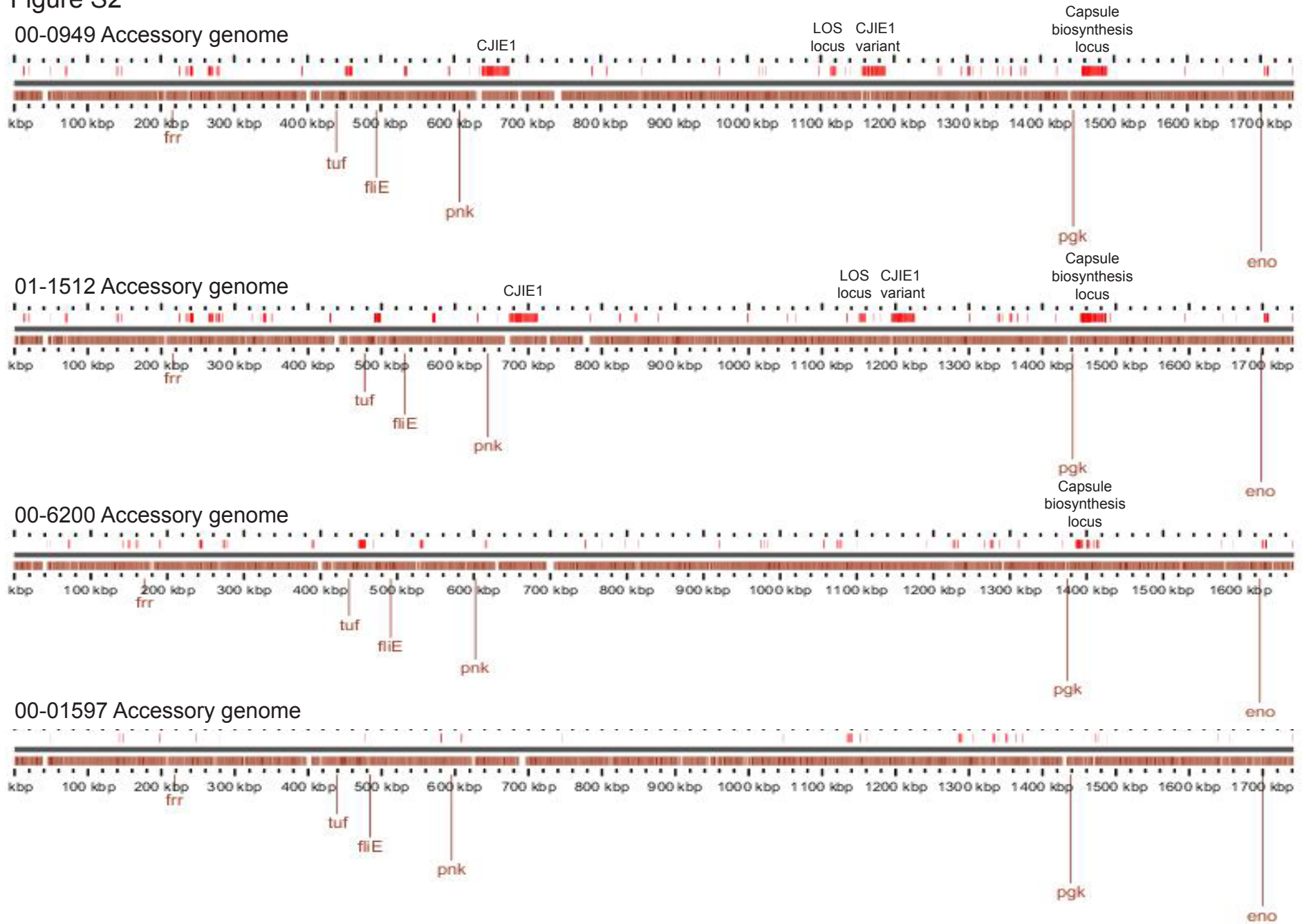

Supplement: S2 Fig — The images were obtained using GView Server, combined, and further annotated using Adobe Illustrator. (PDF) [file pone.0190836.s007.pdf]

Figure S3

00-0949 Unique genome

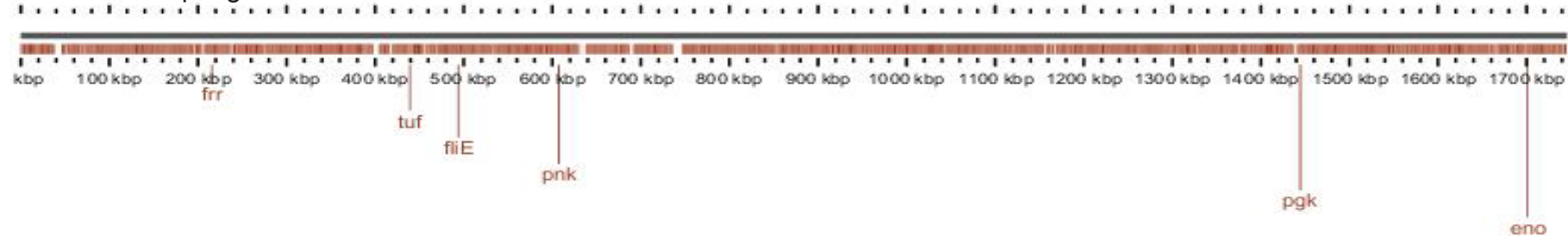

01-1512 Unique genome

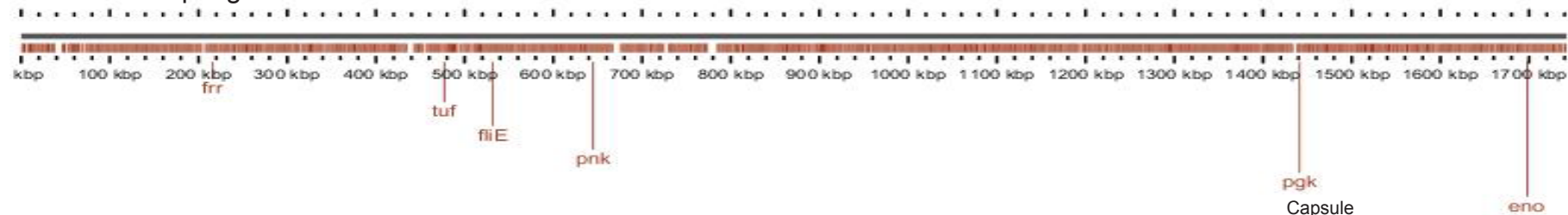

00-6200 Unique genome

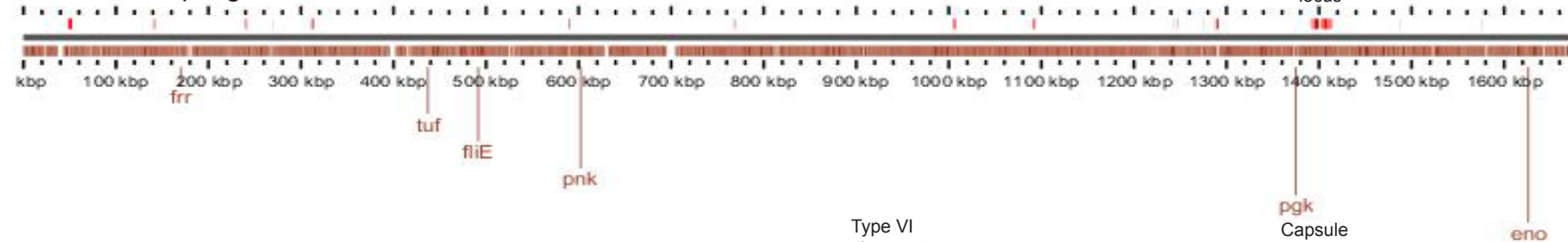

00-01597 Unique genome

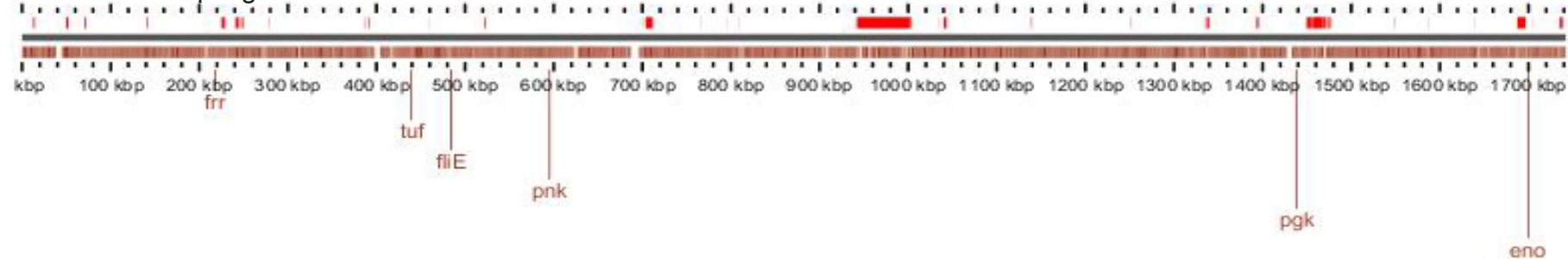

Supplement: S3 Fig — The images were obtained using GView Server, combined, and further annotated using Adobe Illustrator. (PDF) [file pone.0190836.s008.pdf]

Figure S4

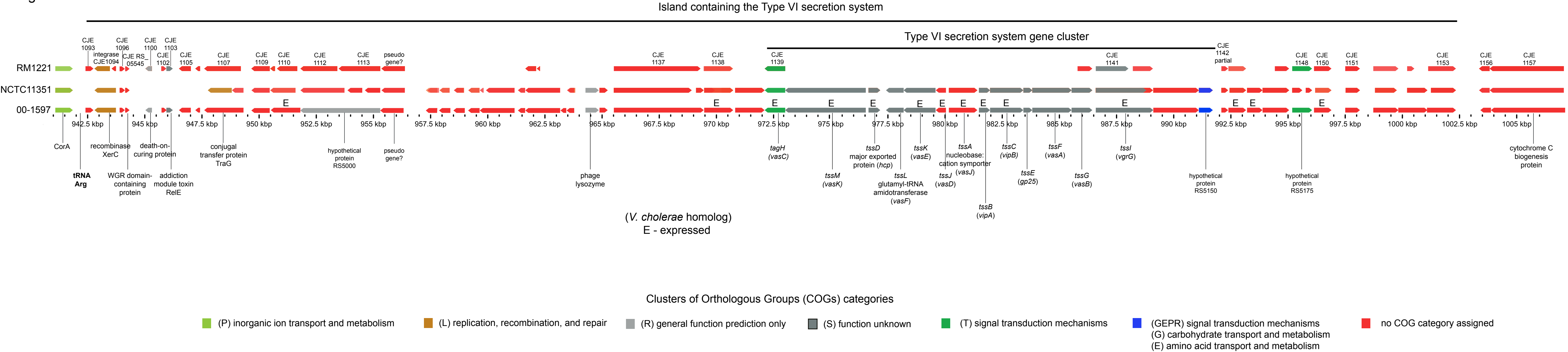

Supplement: S4 Fig — The images were obtained using GView Server, combined, and further annotated using Adobe Illustrator. (PDF) [file pone.0190836.s009.pdf]

Figure S5

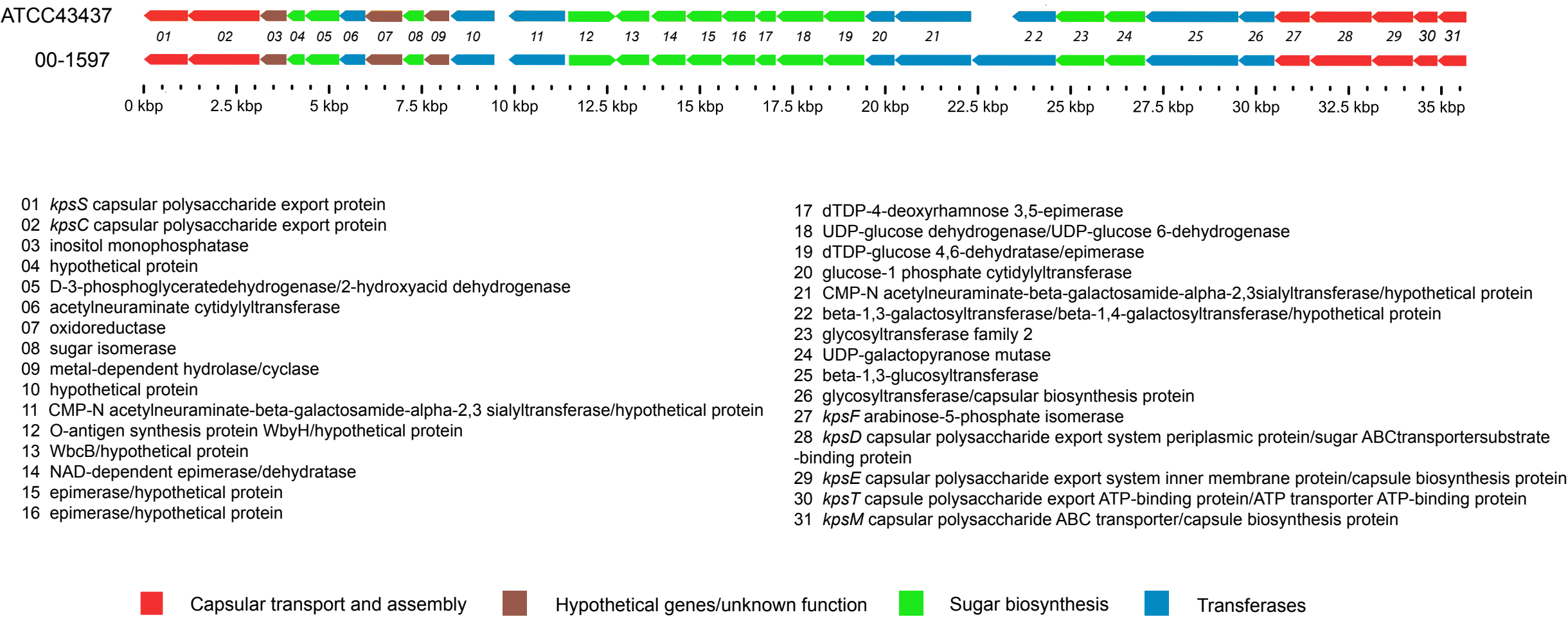

Supplement: S5 Fig — The images were obtained using GView Server, combined, and further annotated using Adobe Illustrator. (PDF) [file pone.0190836.s010.pdf]

Figure S6

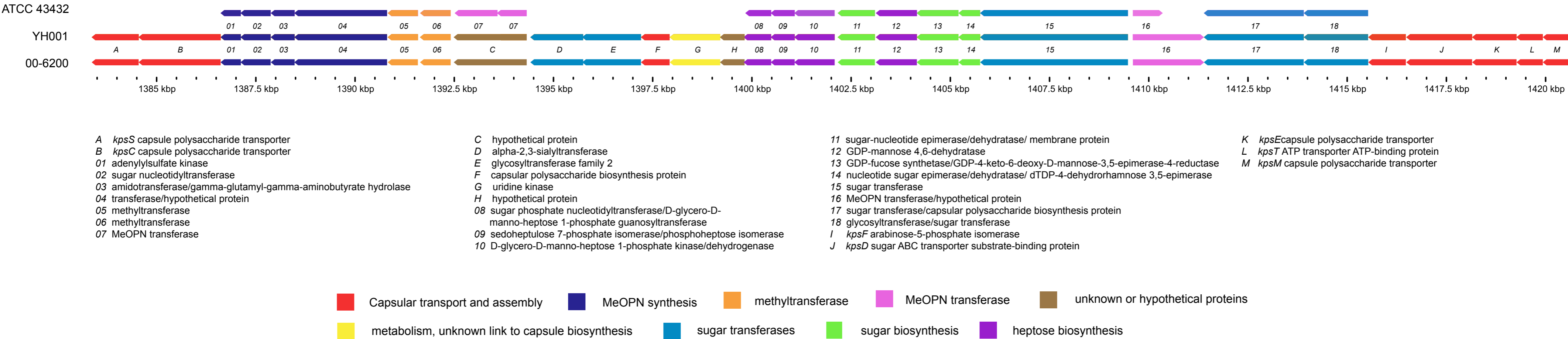

Supplement: S6 Fig — The images were obtained using GView Server, combined, and further annotated using Adobe Illustrator. (PDF) [file pone.0190836.s011.pdf]
