# Supplementary material for: Comparison of genomes and proteomes of four whole genome-sequenced Campylobacter jejuni from different phylogenetic backgrounds
Source: PLoS One. 2018 Jan 2;13(1):e0190836. doi: 10.1371/journal.pone.0190836 (PMC5749857; doi:10.1371/journal.pone.0190836)
Supplement: S1 Table — (DOCX) [file pone.0190836.s012.docx]

S1 Table. Homopolymeric (poly G) tracts in genomes of the four *C. jejuni* isolates.

| **Gene/protein with homolog from NCTC11168 or RM1221** | **Strain** | | | |
| --- | --- | --- | --- | --- |
|  | **00-0949 HS:2** | **01-1512 HS:2** | **00-6200 HS:4,13** | **00-1597 HS:9,37** |
| 104 bp from L-asparaginase ORF (Cj0029) | 38,778 – 11 c | 38,779 – 11 c | 37,022 – 11 c |  |
| Type IIS restriction endonuclease, Cj0031 | 48,799 – **9 g** | 48,800 – **9 g** |  | 48,819 – **9 g** |
| Iron-binding protein, Cj0045 | 65,439 – **10 c** | 65,440 – 11 c (**10**) | 66,441 – 9 c (**10**) | 65,941 – 9 c (**10**) |
| Methyltransferase, Cj0170 | 166,779 – 10 g **(8**) | 166,780 – 10 g (**8**) | 220,401 – 9 c (**8**) | 167,508 – 10 g (**8**) |
| ATP-dependent protease ATP-binding subunit ClpX, Cj0275 | 250,481 – **8 g** | 250,483 – **8 g** | 252,842 – **8 g** |  |
| After ATP/GTP-binding protein, Cj0431 |  |  |  | 397,747 – 10 c |
| Between hypothetical proteins after Cj0563 | 531,778 – 10 g | 569,528 – 10 g |  | 522,055 – 10 g |
| Carbonic anhydrase/motility accessory factor, Cj0617 | 581,983 – **10 g** | 619,733 – **10 g** | 579,732 – 9 g (**10**) | 570,184 – **10 g** |
| Lipoprotein, Cj0638 | 592,762 – 10 g (**9**) | 630,512 – **9 g** |  | 580,963 – 10 g (**9**) |
| In kdpA pseudogene |  |  |  | 622,244 – 11 g |
| Invasion protein CipA, Cj0685 | 681,671 – **9 g** | 719,421 – **9 g** | 639,897 – 8 g (**9**) | 632,119 – 10 g (**9**) |
| After CJE0842 | 738,608 – 11 c | 776,358 – 11 c | 696,829 – 11 c | 689,032 – 11 c |
| Restriction modification enzyme *cjeI*, Cj1051c |  |  | 983,895 – **9 c** |  |
| CJJCF936-1138, CF93-6 (Cj1051c?) |  |  |  | 1,046,633 – **9 c** |
| β-1,3-galactosyltransferase, Cj1139c | 1,115,558 – 9 c (**8**) | 1,153,308 – 9 c (**8**) |  |  |
| Alpha 2,3-sialyltransferase, CJJ81176_1157 (Cj1140?) |  |  |  | 1,136,296 – **9 g** |
| Beta-1,4-N-acetylgalactosaminyltransferase, CJJ81176_1160 |  |  | 1,073,790 – **9 g** | 1,139,452 – **10 g** |
| Acetyltransferase, CJJ81176_1162 |  |  | 1,079,446 – **10 g** |  |
| Hypothetical protein, Cj1145c | 1,121,105 – **9 c** | 1,158,855 – 10 c (**8**) |  |  |
| Between PseC and CJE1487 |  |  |  | 1,326,165 – 10 g |
| Aminopeptidase, Cj1295 | 1,340,196 – 9 g (**10**) | 1,338,807 – 11 g **(10**) | 1,265,242 – 8 g (**9**) |  |
| Aminoglycoside N3´-acetyltransferase, Cj1297 | 1,341,666 – 11 g (**10**) | 1,340,277 – 9 g (**10**) | 1,266,712 – **10 g** |  |
| Carbonic anhydrase, Cj1305 | 1,347,992 - 10 c (**9**) | 1,346,603 – **9 c** |  | 1,333,153 – **9c** |
| Carbonic anhydrase, Cj1306 |  |  | 1,274,281 – **9 c** |  |
| Formyltransferase, CJE1498 |  |  |  | 1,336,507 – 8 g (**9**) |
| 3-oxoacyl-ACP synthase (C. coli) |  |  |  | 1,339,035 – **9 g** |
| Carbonic anhydrase, Cj1310 |  |  | 1,278,420 – **9 c** | 1,340,098 – **9 c** |
| PseD protein/motility accessory factor 4, Cj1318 | 1,354,538 – **9 g** | 1,353,149 – **9 g** | 1,284,967 – **9 g** | 1,346,643 – **9 g** |
| Promoter region, 36 bp upstream of Cj1321 start codon recheck – tellurite resistance protein?? | 1,358,489 – 11 g | 1,357,100 – 11g | 1,290,279 – 11 g | 1,350,600 – 11 g |
| Hypothetical protein, Cj1324 |  |  | 1,291,269 – 9 g (**10**) |  |
| Methyltransferase, Cj1325 | 1,361,366 – 10 g (**9**) | 1,359,977 – 10 g (**9**) | 1,292,700 – **10 g** | 1,353,475 – **9 g** |
| PseD protein/motility accessory factor 4, Cj1335 | 1,371,362 – **9 g** | 1,369,973 – **9 g** | 1,302,698 – **9 g** | 1,363,480 – **9 g** |
| Carbonic anhydrase/motility accessory factor 7, Cj1342 | 1,383,082 **– 9 c** | 1,379,863 – 10 c (**9**) | 1,314,402 – 10 c (**9**) | 1,375,167 – 8 c (**9**) |
| After Cj 1361c |  |  |  | 1,395,473 – 9 c |
| Hypothetical protein not in NCTC11168 |  |  |  | 1,454,942 – **9 c** |
| Epimerase |  |  |  | 1,460,336 – **10 c** |
| Capsular polysaccharide biosynthesis heptosyltransferase HddD |  |  | 1,394,224 – **9 c** |  |
| Hypothetical protein |  |  | 1,399,558 – 10 c (**9**) |  |
| GDP-mannose 4,6-dehydratase,CJJ81176_1426 |  |  | 1,403,310 – **9 c** |  |
| Methyltransferase, Cj1420c | 1, 461,461 – **12 c** | 1,458,242 – 10 c **(9**) | 1,392,008 – **9 c** |  |
| Sugar transferase, Cj1421c | 1,463,672 – **9 c** | 1,460,453 – **9c** | 1,409,696 – **9 g** |  |
| Sugar transferase, Cj1422c | 1,465,604 – **9 c** | 1,462,385 – **9c** |  |  |
| Methyltransferase, Cj1426c | 1,468,583 – **10 c** | 1,465,364 – **10 c** |  |  |
| Hypothetical protein, Cj1429c | 1,471,536 – **10 c** | 1,468,317 – **10 c** |  |  |
| Aminotransferase, Cj1437c | 1, 481,870 – **9 c** | 1,478,608 – **9 c** |  |  |
| Between open reading frames |  |  |  | 1,688,333 – 9 c |
