# Supplementary material for: Comparison of genomes and proteomes of four whole genome-sequenced Campylobacter jejuni from different phylogenetic backgrounds
Source: PLoS One. 2018 Jan 2;13(1):e0190836. doi: 10.1371/journal.pone.0190836 (PMC5749857; doi:10.1371/journal.pone.0190836)
Supplement: S2 Table — (DOCX) [file pone.0190836.s013.docx]

S2 Table. Prophage CJIE4 protein detection in the four *C. jejuni* isolates using comparative iTRAQ proteomic analysis.

| **Protein Identity** | **Homolog in RM1221** | **Non-exclusive peptides** | **Gene identity (LS-BSR)** | | | | **Protein average log_2_ fold change** | | | |
| --- | --- | --- | --- | --- | --- | --- | --- | --- | --- | --- |
|  |  |  | 00-0949 | 01-1512 | 00-6200 | 00-1597 | 00-0949 | 01-1512 | 00-6200 | 00-1597 |
| Integrase PJ17_06545 | CJE1418 | - | 1 | 1 | 1 | 1 | 0.01 | **1.42*** | 0.16 | **-**0.17 |
|  |  | + |  |  |  |  | 0.00 | **1.41*** | 0.07 | -0.19 |
| hypothetical protein PJ17_06555 | CJE1420 | - | 1 | 1 | 0.97 | 0.97 | -0.05 | -1.00 | -0.70 | -0.44 |
|  |  | + |  |  |  |  | 0.01 | -0.77 | -0.40 | -0.44 |
| Emm PJ17_06565 | CJE1422 | - | 1 | 1 | 1 | 1 | **-0.19^†^** | -1.00 | -1.40 | -1.33 |
|  |  | + |  |  |  |  | **-0.20^†^** | -1.03 | -1.53 | -1.36 |
| hypothetical protein PJ19_01890 | NP | - | 1 | 1 | 0.47 | 0.47 | **0.03** | **0.19**^††^ | -1.69 | -1.77 |
|  |  | + |  |  |  |  | **-0.09** | **-0.08**** | -1.16 | -1.14 |
| hypothetical protein PJ17_06595 | CJE1429 | - | 1 | 1 | 1 | 1 | 0.01 | -0.14 | -0.08 | -0.12 |
|  |  | + |  |  |  |  | 0.01 | -0.16 | -0.22 | -0.19 |
| RloG protein PJ17_06600 | CJE1430 | - | 1 | 1 | 1 | 1 | 0.03 | **-**0.35 | -0.35 | -0.44 |
|  |  | + |  |  |  |  | 0.02 | -0.41 | -0.45 | -0.47 |
| hypothetical protein PJ17_06605 | CJE1432 | - | 1 | 1 | 1 | 1 | 0.03 | -0.36 | -0.42 | -1.33 |
|  |  | + |  |  |  |  | 0.06 | -0.37 | -0.48 | -0.35 |
| Cro/CI family transcriptional regulator PJ17_06635 (large indel) | NP | - | 1 | 0.13 | 0 | 1 | **0.00**^#^ | -3.50 | -3.25 | **-0.22** |
|  |  | + |  |  |  |  | **0.00**^#^ | -3.56 | -3.37 | **-0.23** |
| hypothetical protein PJ17_06640 (large indel) | NP | - | 1 | 0 | 0 | 1 | **0.02**^#^ | -4.39 | -3.01 | **-0.43** |
|  |  | + |  |  |  |  | **0.00**^#^ | -4.51 | -3.17 | -**0.47** |
| 3ˊ-5ˊ exonuclease PJ17_06645 (large indel) | NP | - | 1 | 0.11 | 0.11 | 1 | **0.03**^#^ | -4.77 | -3.81 | **-0.44** |
|  |  | + |  |  |  |  | **0.05**^#^ | -4.94 | -4.00 | **-0.46** |
| hypothetical protein PJ17_06650 (large indel) | NP | - | 1 | 0.09 | 0.09 | 1 | **0.01**^#^ | -4.78 | -3.91 | **-0.44** |
|  |  | + |  |  |  |  | **-0.01**^#^ | -4.40 | -3.63 | **-0.46** |
| NTPase KAP PJ17_06655 (large indel) | NP | - | 1 | 0.1 | 0.03 | 1 | **-0.02**^#^ | -4.58 | -3.83 | **-0.40** |
|  |  | + |  |  |  |  | **-0.00**^#^ | -4.65 | -3.92 | **-0.38** |
| hypothetical protein PJ18_06440 (alternate large indel) | CJE1439 | - | 0.8 | 1 | 1 | 0.8 | -0.01 | **4.50** | **2.73** | -0.52 |
|  |  | + |  |  |  |  | 0.02 | **4.52** | **2.69** | -0.49 |
| peptidase S24 PJ18_06445 (alternate large indel) | CJE1440 | - | 0.11 | 1 | 1 | 0.07 | -0.13 | **3.71**^§^ | **2.81** | 0.00 |
|  |  | + |  |  |  |  | -0.11 | **3.74**^§^ | **2.76** | 0.01 |
| endonuclease PJ18_06450 (alternate large indel) | CJE1441 | - | 0.67 | 1 | 1 | 0.67 | -0.02 | **3.98**^§^ | **3.47** | 0.09 |
|  |  | + |  |  |  |  | -0.16^§^ | **3.81**^§^ | **3.21** | 0.05 |
| hypothetical protein PJ18_06455 (alternate large indel) | CJE1442 | - | 0 | 1 | 1 | 0 | -0.39 | **4.91**^§§^ | **4.74** | 0.48 |
|  |  | + |  |  |  |  | -0.47 | **4.92**^§§^ | **4.70** | 0.01 |
| hypothetical protein PJ17_06660 | CJE1444 | - | 0.99 | 1 | 1 | 1 | **0.06** | **0.02**** | -2.87 | -2.73 |
|  |  | + |  |  |  |  | **0.05** | **-0.02**** | -2.97 | -2.74 |
| hypothetical protein PJ16_07060 | CJE1447 | - | 1 | 1 | 1 | 1 | **0.07**^†^ | -2.89 | -1.92 | -1.90 |
|  |  | + |  |  |  |  | **0.12**^†^ | -3.02 | -2.07 | -1.88 |
| hypothetical protein PJ17_06675 | CJE1448 | - | 0.98 | 1 | 0.97 | 1 | **-0.04**^†^ | -2.91 | -2.52 | -2.18 |
|  |  | + |  |  |  |  | **0.05*** | -2.83 | -2.50 | -2.21 |
| hypothetical protein PJ19_01755 (PJ16_07085) | CJE1452 | - | 1 | 1 | 1 | 1 | **0.13**^†^ | -3.43 | -3.19 | -2.83 |
|  |  | + |  |  |  |  | **-0.08*** | -3.12 | -2.56 | -2.52 |
| hypothetical protein PJ17_06695 | CJE1452 | - | 1 | 1 | 1 | 1 | ND | ND | ND | ND |
|  |  | + |  |  |  |  | **-0.03*** | -2.98 | -2.22 | -2.30 |
| capsid protein PJ17_06720 | CJE1458 | - | 1 | 1 | 1 | 1 | **0.05*** | -3.44 | -2.80 | -3.02 |
|  |  | + |  |  |  |  | **0.07*** | -3.52 | -2.92 | -3.09 |
| DNA repair protein PJ17_06750 | CJE1465 | - | 1 | 1 | 1 | 1 | **-0.40**^‡^ | -3.09 | -1.91 | -2.69 |
|  |  | + |  |  |  |  | **-0.42*** | -3.14 | -2.00 | -2.71 |
| hypothetical protein PJ18_06555 | CJE1466 | - | 1 | 1 | 0.99 | 1 | -0.02^‡^ | 0.24 | **1.73**^†^ | 0.05 |
|  |  | + |  |  |  |  | **0.04*** | -3.68 | -2.33 | -2.73 |
| hypothetical protein PJ19_01695 | CJE1466 | - | 1 | 1 | 0.99 | 1 | **0.29**^‡^ | -4.05 | -4.56 | -3.71 |
|  |  | + |  |  |  |  | **0.06*** | -4.06 | -3.24 | -3.18 |
| hypothetical protein PJ18_06560 (unique to 00-6200) | NP | - | 0 | 0 | 1 | 0 | 0.03 | 0.01 | **3.85*** | 0.09 |
|  |  | + |  |  |  |  | 0.02 | -0.03 | **3.76*** | 0.07 |
| toxin-antitoxin system protein PJ17_06775 | CJE1470 | - | 1 | 1 | 0.99 | 1 | ND | ND | ND | ND |
|  |  | + |  |  |  |  | 0.03 | -0.02 | -0.00 | **-**0.28 |

Isolate 00-0949 was used as the reference strain for iTRAQ analysis; NP – not present; ND – not detected

Statistical analysis using Mann-Whitney test with Benjamini-Hochberg correction, isolate tagged vs the three other isolates: ^†^*P* <0.05, ^‡^ *P* <0.001, **P* <0.0001; 00-0949 and 01-1512 vs the other two isolates: ^††^*P* < 0.01, ***P* <0.0001; 00-0949 and 00-1597 vs the other two isolates: ^#^*P* < 0.0001; 01-1512 and 00-6200 vs the other two isolates: ^§§^*P* < 0.01, ^§^*P* < 0.0001
