# Supplementary material for: Comparison of genomes and proteomes of four whole genome-sequenced Campylobacter jejuni from different phylogenetic backgrounds
Source: PLoS One. 2018 Jan 2;13(1):e0190836. doi: 10.1371/journal.pone.0190836 (PMC5749857; doi:10.1371/journal.pone.0190836)
Supplement: S3 Table — (DOCX) [file pone.0190836.s014.docx]

S3 Table. CJIE1 and CJIE1 variant prophage proteins and detection of proteins in 4-plex iTRAQ comparative proteomics experiments.

| **RM1221 homolog** | **Protein identity** | **CJIE1** | **CJIE1**  **variant** | **CJIE4** | **Non-exclusive peptides** | **Average log_2_ fold change** | | | |
| --- | --- | --- | --- | --- | --- | --- | --- | --- | --- |
|  |  |  |  |  |  | **00-0949** | **01-1512** | **00-6200** | **00-1597** |
| CJE0213 | hypothetical protein PJ16_03380 | + | - | - |  | ND | ND | ND | ND |
| CJE0215 | repressor PJ16_03380 | + | + | - | -^1^ | 0.03 | -0.81 | ND | -3.49 |
|  |  |  |  |  | + | **-0.13** | **-0.42**^‡^ | -2.61 | -2.52 |
| CJE0215 | repressor PJ16_06175 | + | + | - | -^1^ | **0.04** | **-0.07** | -1.37 | -1.55 |
|  |  |  |  |  | + | **0.20** | **-0.18**^‡^ | -2.21 | -2.54 |
| CJE0216 | hypothetical protein PJ16_03385 | + | + | - | -^1^ | **0.01** | **-0.10** | -1.11 | -2.15 |
|  |  |  |  |  | + | **0.16** | **0.04**^‡^ | -3.37 | -3.67 |
|  | hypothetical protein PJ16_06180 | + | + | - | - | ND | ND | ND | ND |
|  |  |  |  |  | + | **0.05** | **-0.08**^‡^ | -3.72 | -4.01 |
| CJE0217 | hypothetical protein PJ16_03390, PJ16_06185 | + | + | - | - | **-0.07** | **-0.05**^§^ | -2.96 | -3.65 |
|  |  |  |  |  | + | **0.06** | **-0.08**^§^ | -3.04 | -3.66 |
| CJE0219 | hypothetical protein PJ16_03395, PJ16_06190 | + | + | - |  | ND | ND | ND | ND |
| CJE0220 | DNA adenine methylase PJ16_03400, PJ16_06195 | + | + | - |  | ND | ND | ND | ND |
| CJE0221 | virion morphogenesis protein PJ16_03405 | + | - | - |  | ND | ND | ND | ND |
| CJE0222 | tail tape measure protein PJ16_03410 | + | - | - |  | ND | ND | ND | ND |
| CJE0223 | hypothetical protein PJ16_03415 | + | - | - |  | ND | ND | ND | ND |
| CJE0225 | hypothetical protein PJ16_03420 | + | - | - |  | ND | ND | ND | ND |
| CJE0226 | tail protein PJ16_03425 | + | - | - |  | ND | ND | ND | ND |
|  | phage tail protein PJ16_06200 | - | + | - |  | ND | ND | ND | ND |
|  | tail protein PJ16_06205 | - | + | - |  | ND | ND | ND | ND |
|  | tail protein PJ16_06210 | - | + | - |  | ND | ND | ND | ND |
|  | tail tape measure protein PJ16_06215 | - | + | - |  | ND | ND | ND | ND |
|  | membrane protein PJ16_06220 | - | + | - |  | ND | ND | ND | ND |
|  | hypothetical protein PJ16_06225 | - | + | - |  | ND | ND | ND | ND |
|  | tail protein PJ16_06230 | - | + | - |  | ND | ND | ND | ND |
| CJE0227 | tail sheath protein PJ16_03430, PJ16_06235 | + | + | - | - | ND | ND | ND | ND |
|  |  |  |  |  | + | **0.16** | **0.04** | -3.37 | -3.67 |
| CJE0228 | hypothetical protein PJ16_03435, PJ16_06240 | + | + | - | - | **-0.22** | **-0.27** | -1.87 | -0.26 |
|  |  |  |  |  | + | **-0.24** | **-0.29** | -2.01 | -0.25 |
| CJE0229 | hypothetical protein PJ16_03440, PJ16_06245 | + | + | + | - | ND | ND | ND | ND |
|  |  |  |  |  | + | **-0.07**^††^ | -1.73 | -2.20 | -2.17 |
| CJE0230 | hypothetical protein PJ16_03445, PJ16_06250 | + | + | - |  | ND | ND | ND | ND |
| CJE0231 | hypothetical protein PJ16_03450, PJ16_06255 | + | + | - |  | ND | ND | ND | ND |
| CJE0232 | hypothetical protein PJ16_03455, PJ16_06260 | + | + | - |  | ND | ND | ND | ND |
| CJE0233 | baseplate assembly protein PJ16_03460, PJ16_06265 | + | + | - |  | ND | ND | ND | ND |
| CJE0234 | baseplate assembly protein PJ16_03465, PJ16_06270 | + | + | - |  | ND | ND | ND | ND |
| CJE0235 | membrane protein PJ16_03470, PJ16_06275 | + | + | - |  | ND | ND | ND | ND |
| CJE0236 | baseplate assembly protein PJ16_03475, PJ16_06280 | + | + | - |  | ND | ND | ND | ND |
|  | resolvase PJ16_06285 | - | + | - |  | ND | ND | ND | ND |
|  | hypothetical protein PJ16_06290 | - | + | - |  | ND | ND | ND | ND |
|  | head protein PJ16_06295 | - | + | - |  | ND | ND | ND | ND |
|  | hypothetical protein PJ16_06300 | - | + | - |  | ND | ND | ND | ND |
|  | hypothetical protein PJ16_06305 | - | + | - |  | ND | ND | ND | ND |
|  | hypothetical protein PJ16_06310 | - | + | - |  | ND | ND | ND | ND |
| CJE0237 | hypothetical protein PJ16_03480, PJ16_06335 | + | + | - |  | ND | ND | ND | ND |
| CJE0238 | membrane protein PJ16_03485, PJ16_06330 | + | + | - |  | ND | ND | ND | ND |
| CJE0239 | hypothetical protein PJ16_03490, PJ16_06325 | + | + | - |  | ND | ND | ND | ND |
| CJE0240 | hypothetical protein PJ16_03495, PJ16_06320 | + | + | - |  | ND | ND | ND | ND |
| CJE0241 | peptidase M15 family protein PJ16_03500, PJ16_06315 | + | + | - |  | ND | ND | ND | ND |
|  | hypothetical protein PJ16_06340 | - | + | - |  | ND | ND | ND | ND |
|  | hypothetical protein PJ16_06345 | - | + | - |  | ND | ND | ND | ND |
|  | hypothetical protein PJ16_06350 | - | + | - |  | ND | ND | ND | ND |
|  | hypothetical protein PJ16_06355 | - | + | - |  | ND | ND | ND | ND |
|  | transcriptional regulator PJ16_06360 | - | + | - |  | ND | ND | ND | ND |
|  | hypothetical protein PJ16_06365 | - | + | - |  | ND | ND | ND | ND |
|  | hypothetical protein PJ16_06370 | - | + | - |  | ND | ND | ND | ND |
| CJE0243 | hypothetical protein PJ16_03505 | + | - | - |  | ND | ND | ND | ND |
| CJE0244 | hypothetical protein PJ16_03510 | + | - | - |  | ND | ND | ND | ND |
| CJE0245 | hypothetical protein PJ16_03515 | + | - | - |  | ND | ND | ND | ND |
| CJE0246 | hypothetical protein PJ16_03520 | + | - | - | - | **-0.13** | **-0.14**^†^ | -2.23 | -2.21 |
|  |  |  |  |  | + | **-0.12** | **-0.14**^†^ | -2.32 | -2.23 |
| CJE0247 | hypothetical protein PJ16_03525 | + | - | - |  | ND | ND | ND | ND |
| CJE0248 | hypothetical protein PJ16_03530 | + | - | - |  | ND | ND | ND | ND |
| CJE0249 | hypothetical protein PJ16_03535 | + | - | - |  | ND | ND | ND | ND |
| CJE0250 | hypothetical protein PJ16_03540 | + | - | - |  | ND | ND | ND | ND |
| CJE0251 | hypothetical protein PJ16_03545 | + | - | - |  | ND | ND | ND | ND |
| CJE0252 | tail protein PJ16_03550 | + | - | - |  | ND | ND | ND | ND |
| CJE0253 | tail protein PJ16_03555 | + | - | - |  | ND | ND | ND | ND |
| CJE0254 | tail protein PJ16_03560 | + | - | - |  | ND | ND | ND | ND |
|  | hypothetical protein PJ16_03565 | + | - | - | - | **0.06** | **0.01** | -3.75 | -3.76 |
|  |  |  |  |  | + | **0.03** | **-0.04** | -3.88 | -3.82 |
|  | hypothetical protein PJ16_03570 | + | - | - | - | **0.00** | **0.08*** | -2.60 | -3.93 |
|  |  |  |  |  | + | **0.03** | **0.09*** | -3.27 | -4.04 |
| CJE0255 | DNA-binding protein PJ16_03575 | + | - | - |  | ND | ND | ND | ND |
| CJE0256 | deoxyribonuclease PJ16_03580 | + | - | - | - | **0.00** | **0.07*** | -2.46 | -2.45 |
|  |  |  |  |  | + | **0.01** | **0.04*** | -2.54 | -2.46 |
| CJE0257 | hypothetical protein PJ16_03585 | + | - | - | - | **-0.29** | **-0.19**^§^ | -3.42 | -3.48 |
|  |  |  |  |  | + | **-0.08** | **-0.14**^§^ | -3.82 | -3.79 |
| CJE0258 | hypothetical protein PJ16_03590 | + | - | - |  | ND | ND | ND | ND |
| CJE0259 | hypothetical protein PJ16_03595 | + | - | - |  | ND | ND | ND | ND |
| CJE0260 | hypothetical protein PJ16_03600 | + | - | - |  | ND | ND | ND | ND |
| CJE0261 | hypothetical protein PJ16_03605 | + | - | - |  | ND | ND | ND | ND |
| CJE0262 | hypothetical protein PJ16_03610, PJ16_06375 | + | + | - | - | **0.02** | **0.02*** | -2.35 | -2.03 |
|  |  |  |  |  | + | **0.00** | **-0.09*** | -3.63 | -3.29 |
| CJE0263 | hypothetical protein PJ16_03615, PJ16_06380 | + | + | - | - | **0.02** | **0.20*** | -2.93 | -2.90 |
|  |  |  |  |  | + | **0.04** | **0.23*** | -3.29 | -3.29 |
| CJE0264 | hypothetical protein PJ16_03620, PJ16_06385 | + | + | - |  | ND | ND | ND | ND |
| CJE0265 | Host-nuclease inhibitor protein Gam PJ16_03625, PJ16_06390 | + | + | - |  | ND | ND | ND | ND |
|  | hypothetical protein PJ16_06395 | - | + | - |  | ND | ND | ND | ND |
| CJE0266 | hypothetical protein PJ16_06400 | - | + | - | - | **-0.51** | -2.39 | ND | -3.28 |
|  |  |  |  |  | + | **-0.66** | -1.48 | -3.44 | -3.18 |
| CJE0266 | hypothetical protein PJ19_03895 | + | - | - | - | 0.01 | 0.36 | 0.51 | 0.21 |
|  |  |  |  |  | + | **0.06** | **0.12**^†^ | -1.62 | -1.72 |
|  | hypothetical protein PJ16_06405 | - | + | - |  | ND | ND | ND | ND |
| CJE0267 | hypothetical protein PJ16_03635, PJ16_06410 | + | + | - |  | ND | ND | ND | ND |
| CJE0268 | RNA polymerase sigma70 PJ16_03640, PJ16_06415 | + | + | - |  | ND | ND | ND | ND |
| CJE0269 | bacteriocin/transposition protein B PJ16_03645 | + | - | - |  | ND | ND | ND | ND |
| CJE0270 | integrase/transposition protein A PJ16_03650 | + | - | - |  | ND | ND | ND | ND |
|  | bacteriocin/transposition protein B PJ16_06420 | - | + | - |  | ND | ND | ND | ND |
|  | integrase/transposition protein A PJ16_06425 | - | + | - |  | ND | ND | ND | ND |
|  | hypothetical protein PJ16_06430 | - | + | - |  | ND | ND | ND | ND |
|  | DNA-binding protein PJ16_06435 | - | + | - | - | **0.02** | **0.12*** | -3.36 | -3.67 |
|  |  |  |  |  | + | **-0.20** | **0.12*** | -2.08 | -1.98 |
| CJE0271 | hypothetical protein PJ16_03655 | + | - | - |  | ND | ND | ND | ND |
| CJE0272 | transcriptional regulator PJ16_03660 | + | - | - | - | **0.02** | **0.32*** | -2.47 | -2.43 |
|  |  |  |  |  | + | **0.01** | **0.31*** | -2.87 | -2.94 |
| CJE0273 | hypothetical protein PJ16_03665 | + | - | - | - | **0.64** | **0.81*** | -2.54 | -2.46 |
|  |  |  |  |  | + | **0.02** | **0.20*** | -2.14 | -2.27 |

^1^detected in only one or two replicate experiments

Statistical analysis using Mann-Whitney test with Benjamini-Hochberg correction, 00-0949 and 01-1512 vs the other two isolates: ^†^*P* <0.05, ^§^*P* <0.01, ^‡^*P* <0.001, **P* <0.0001; 00-0949 and 01-1512 vs the other three isolates: ^††^*P* <0.05
