# Supplementary material for: Comparison of genomes and proteomes of four whole genome-sequenced Campylobacter jejuni from different phylogenetic backgrounds
Source: PLoS One. 2018 Jan 2;13(1):e0190836. doi: 10.1371/journal.pone.0190836 (PMC5749857; doi:10.1371/journal.pone.0190836)
Supplement: S4 Table — (DOCX) [file pone.0190836.s015.docx]

S4 Table. Detection of proteins in the 00-1597 Type VI secretion island using comparative 4-plex iTRAQ proteomic analysis.

| **Protein Identity** | **RM1221**  **homolog** | **Non-exclusive peptides** | **Gene identity (LS-BSR)** | | | | **Protein average log_2_ fold change** | | | |
| --- | --- | --- | --- | --- | --- | --- | --- | --- | --- | --- |
|  |  |  | **00-0949** | **01-1512** | **00-6200** | **00-1597** | **00-0949** | **01-1512** | **00-6200** | **00-1597** |
| hypothetical protein PJ17_04995 | CJE1110 | - | 0 | 0 | 0 | 1 | 0.01 | -0.12 | 0.32 | **2.45**^‡^ |
|  |  | + |  |  |  |  | -0.04 | -0.22 | 0.32 | **2.31**^‡^ |
| hypothetical protein PJ17_05070 | CJE1138 | - | 0 | 0 | 0 | 1 | 2.21 | 2.49 | 2.38 | **4.30** |
|  |  | + |  |  |  |  | 0.07 | 0.23 | 0.58 | **2.79**^‡^ |
| hypothetical protein PJ17_05075 |  | - | 0 | 0 | 0 | 1 | ND | ND | ND | ND |
|  |  | + |  |  |  |  | -0.01 | -0.18 | 0.17 | **2.47**^‡^ |
| hypothetical protein PJ17_05080 (VasC homolog) | CJE1139 | - | 0.06 | 0.06 | 0.06 | 1 | 0.00 | -0.36 | -0.48 | **4.04*** |
|  |  | + |  |  |  |  | 0.01 | -0.38 | 0.42 | **4.08*** |
| hypothetical protein PJ17_05085 (VasK homolog) |  | - | 0 | 0 | 0 | 1 | 0.01 | -0.42 | 0.28 | **3.80*** |
|  |  | + |  |  |  |  | 0.03 | 0.91 | 1.08 | **4.54*** |
| major exported protein PJ17_05090 (Hcp) |  | - | 0 | 0 | 0 | 1 | -0.04 | -0.45 | 0.21 | **3.85*** |
|  |  | + |  |  |  |  | -0.04 | -0.55 | 0.83 | **5.05*** |
| type VI secretion protein PJ17_05100 (VasE homolog) |  | - | 0.04 | 0.04 | 0.04 | 1 | -0.01 | -0.24 | 0.63 | **4.44*** |
|  |  | + |  |  |  |  | -0.01 | -0.30 | 0.57 | **4.54*** |
| type VI secretion protein PJ17_05105 (VasD homolog) |  | - | 0 | 0 | 0 | 1 | -0.02 | -0.69 | 0.25 | **3.88*** |
|  |  | + |  |  |  |  | -0.01 | -0.73 | 0.17 | **3.90*** |
| nucleobase:cation symporter PJ17_05110 (VasJ homolog) |  | - | 0 | 0 | 0.4 | 1 | -0.10 | -0.71 | 0.24 | **3.87*** |
|  |  | + |  |  |  |  | -0.08 | -0.75 | 0.14 | **3.91*** |
| type VI secretion protein PJ17_05115 (VipA homolog) |  | - | 0 | 0 | 0 | 1 | 0.00 | -0.65 | 0.57 | **4.28*** |
|  |  | + |  |  |  |  | -0.02 | -0.68 | 0.49 | **4.28*** |
| type VI secretion protein PJ17_05120 (VipB homolog) |  | - | 0.03 | 0.03 | 0.03 | 1 | -0.02 | -1.00 | 0.68 | **4.92*** |
|  |  | + |  |  |  |  | -0.04 | -1.07 | 0.59 | **4.93*** |
| type VI secretion protein VgrG PJ17_05140 | CJE1141 | - | 0.02 | 0.02 | 0.02 | 1 | -0.04 | -0.55 | 0.39 | **3.51*** |
|  |  | + |  |  |  |  | -0.03 | -0.59 | 0.31 | **3.54*** |
| hypothetical protein PJ17_05160 |  | - | ND | ND | ND | ND | ND | ND | ND | ND |
|  |  | + |  |  |  |  | -0.02 | -0.09 | 0.42 | **3.01**^†^ |
| hypothetical protein PJ17_05165 |  | - | 0.07 | 0.07 | 0.07 | 1 | -0.04 | 0.10 | 0.40 | **3.21** |
|  |  | + |  |  |  |  | -0.02 | 0.09 | 034 | **3.28** |
| hypothetical protein PJ17_05180 | CJE1150 | - | 0.07 | 0.07 | 0.06 | 1 | 0.01 | -0.67 | 0.09 | **2.45**^‡^ |
|  |  | + |  |  |  |  | -0.03 | -0.68 | -0.16 | **2.45**** |
| hypothetical protein PJ17_05200 | CJE1153 | - | 0 | 0 | 0 | 1 | 0.01 | 0.27 | 1.00 | **4.43**^†^ |
|  |  | + |  |  |  |  | -0.02 | 0.23 | 0.95 | **4.48**^†^ |

Isolate 00-0949 was used as the reference strain for iTRAQ analysis; NP – not present; ND – not detected/no data

Statistical analysis using Mann-Whitney test with Benjamini-Hochberg correction, 00-1597 vs the three other isolates: ^†^*P* <0.05, ^‡^*P* <0.01, ***P* <0.001, **P* <0.0001;
