# Supplementary material for: Comparison of genomes and proteomes of four whole genome-sequenced Campylobacter jejuni from different phylogenetic backgrounds
Source: PLoS One. 2018 Jan 2;13(1):e0190836. doi: 10.1371/journal.pone.0190836 (PMC5749857; doi:10.1371/journal.pone.0190836)
Supplement: S5 Table — (DOCX) [file pone.0190836.s016.docx]

S5 Table. Detection of proteins in the 00-1597 HS:9 CPB cluster using comparative 4-plex iTRAQ proteomic analysis.

| **Protein Identity** | **Non-exclusive peptides** | **Gene identity (LS-BSR)** | | | | **Protein average log_2_ fold change** | | | |
| --- | --- | --- | --- | --- | --- | --- | --- | --- | --- |
|  |  | **00-0949** | **01-1512** | **00-6200** | **00-1597** | **00-0949** | **01-1512** | **00-6200** | **00-1597** |
| *kpsS* capsule biosynthesis protein PJ17_07435 | - | 1 | 1 | 0.99 | 0.88 | ND | ND | ND | ND |
|  | + |  |  |  |  | ND | ND | ND | ND |
| *kpsC* capsule biosynthesis protein PJ17_07440 | - | 1 | 1 | 0.95 | 0.85 | 0.05 | -1.55 | -1.55 | **1.52** |
|  | + |  |  |  |  | -0.066 | -0.16 | 0.12 | **0.90**^‡^ |
| inositol monophosphatase PJ17_07445 | - | 0.15 | 0.15 | 0.15 | 1 | 0.01 | 0.37 | 0.54 | **3.99*** |
|  | + |  |  |  |  | 0.01 | -0.40 | 0.47 | **3.98*** |
| hypothetical protein PJ17_07450 | - | 0 | 0 | 0 | 1 | -0.14 | -1.01 | 1.27 | **5.08*** |
|  | + |  |  |  |  | -0.21 | -1.13 | 1.22 | **5.10*** |
| 2-hydroxyacid dehydrogenase PJ17_07455 | - | 0.21 | 0.21 | 0.21 | 1 | -0.04 | -0.69 | 0.87 | **4.77*** |
|  | + |  |  |  |  | -0.06 | -0.75 | 0.81 | **4.81*** |
| acetylneuraminate cytidylyltransferase PJ17_07460 | - | 0.16 | 0.16 | 0.17 | 1 | -0.03 | -0.32 | 0.48 | **4.68*** |
|  | + |  |  |  |  | -0.03 | -0.37 | 0.41 | **4.72*** |
| oxidoreductase PJ17_07465 | - | 0.12 | 0.12 | 0.12 | 1 | -0.03 | -0.77 | 0.51 | **4.40*** |
|  | + |  |  |  |  | -0.01 | -0.78 | 0.45 | **4.44*** |
| sugar isomerase PJ17_07470 | - | 0.24 | 0.24 | 0.25 | 1 | 0.01 | -0.18 | 1.10 | **6.24*** |
|  | + |  |  |  |  | 0.00 | -1.09 | 1.01 | **6.37*** |
| cyclase PJ17_07475 | - | 0.07 | 0.07 | 0 | 1 | 0.57 | 0.93 | 0.92 | **4.10*** |
|  | + |  |  |  |  | 0.78 | 1.35 | 1.07 | **4.21**^‡^ |
| hypothetical protein PJ17_07480 | - | 0.12 | 0.12 | 0.17 | 1 | ND | ND | ND | ND |
|  | + |  |  |  |  | ND | ND | ND | ND |
| hypothetical protein PJ17_07490 | - | 0.12 | 0.12 | 0.17 | 1 | 0.00 | -0.74 | 0.54 | **4.30*** |
|  | + |  |  |  |  | -0.02 | -0.85 | -0.54 | **4.35*** |
| hypothetical protein PJ17_07495 | - | 0.06 | 0.06 | 0 | 1 | -0.02 | -0.47 | 0.07 | **2.71*** |
|  | + |  |  |  |  | -0.03 | -0.52 | -0.02 | **2.68*** |
| hypothetical protein /dehydratase PJ17_07500 | - | 0.12 | 0.12 | 0.12 | 1 | ND | ND | ND | ND |
|  | + |  |  |  |  | ND | ND | ND | ND |
| hypothetical protein PJ17_07505 | - | 0.11 | 0.11 | 0.11 | 1 | ND | ND | ND | ND |
|  | + |  |  |  |  | ND | ND | ND | ND |
| hypothetical protein PJ17_07510 | - | 0.1 | 0.1 | 0.1 | 1 | 0.00 | -0.82 | 0.60 | **4.28*** |
|  | + |  |  |  |  | 0.00 | -0.87 | 0.53 | **4.29*** |
| hypothetical protein PJ17_07515 | - | 0 | 0 | 0 | 1 | 0.02 | -0.54 | 0.31 | **4.83*** |
|  | + |  |  |  |  | 0.03 | -0.73 | 0.32 | **4.92*** |
| dTDP-4-deoxyrhamnose 3,5-epimerase PJ17_07520 | - | 0.18 | 0.18 | 0 | 1 | -0.03 | -0.65 | 0.42 | **4.47*** |
|  | + |  |  |  |  | -0.03 | -0.69 | 0.35 | **4.50*** |
| UDP-glucose-6-dehydrogenase PJ17_07525 | - | 0.14 | 0.14 | 0 | 1 | -0.06 | -0.63 | 0.75 | **4.97*** |
|  | + |  |  |  |  | -0.05 | -0.67 | 0.69 | **5.03*** |
| epimerase PJ17_07530 | - | 0.12 | 0.12 | 0.11 | 1 | -0.07 | -0.81 | 0.90 | **5.62*** |
|  | + |  |  |  |  | -0.07 | -1.04 | 0.84 | **5.69*** |
| glucose-1-phosphate cytidylyltransferase PJ17_07535 | - | 0.12 | 0.12 | 0.12 | 1 | -0.05 | -0.72 | 0.85 | **5.44*** |
|  | + |  |  |  |  | -0.04 | -1.14 | 0.80 | **5.46*** |
| hypothetical protein PJ17_07540 | - | 0.09 | 0.09 | 0.15 | 1 | -0.09 | -0.72 | 0.77 | **4.70*** |
|  | + |  |  |  |  | -0.11 | -0.86 | 0.72 | **4.71*** |
| hypothetical protein PJ17_07545 | - | 0.3 | 0.3 | 0.28 | 1 | ND | ND | ND | ND |
|  | + |  |  |  |  | ND | ND | ND | ND |
| glycosyltransferase family 2 PJ17_07550 | - | 0.49 | 0.49 | 0.09 | 1 | 0.00 | -0.38 | 0.65 | **4.37*** |
|  | + |  |  |  |  | -0.02 | -0.51 | 0.56 | **4.42*** |
| UDP-galactopyranose mutase PJ17_07555 | - | 0.8 | 0.8 | 0.04 | 1 | -0.02 | -0.37 | 0.87 | **4.81*** |
|  | + |  |  |  |  | -0.02 | -0.47 | 0.58 | **4.51*** |
| capsular biosynthesis protein PJ17_07560 | - | 0.05 | 0.05 | 0.57 | 1 | 0.01 | -0.35 | 0.12 | **4.01** |
|  | + |  |  |  |  | -0.04 | -0.12 | 0.92 | **3.28** |
| capsular biosynthesis protein PJ17_07565 | - | 0.11 | 0.11 | 0.11 | 1 | 0.03 | -0.45 | 0.16 | **3.77**^‡^ |
|  | + |  |  |  |  | 0.01 | -0.54 | 0.05 | **3.24**^‡^ |
| *kpsF* arabinose-5-phosphate isomerase PJ17_07570 | - | 1 | 1 | 1 | 0.97 | 0.02 | 0.01 | 0.15 | **4.55*** |
|  | + |  |  |  |  | 0.01 | -0.05 | 0.06 | **1.58** |
| *kpsD* sugar ABC transporter substrate-binding protein PJ17_07575 | - | 1 | 1 | 1 | 0.99 | -0.29 | -0.10 | 1.43 | **6.12**^†^ |
|  | + |  |  |  |  | 0.02 | -0.02 | 0.07 | 0.23 |
| *kpsE* capsule biosynthesis protein PJ17_07580 | - | 1 | 1 | 0.98 | 1 | ND | ND | ND | ND |
|  | + |  |  |  |  | ND | ND | ND | ND |
| *kpsT* ABC transporter ATP-binding protein PJ17_07585 | - | 1 | 1 | 1 | 0.98 | ND | ND | ND | ND |
|  | + |  |  |  |  | ND | ND | ND | ND |
| *kpsM* capsule biosynthesis protein PJ17_07590 | - | 1 | 1 | 0.97 | 0.97 | ND | ND | ND | ND |
|  | + |  |  |  |  | ND | ND | ND | ND |

Isolate 00-0949was used as the reference strain for iTRAQ analysis; NP – not present; ND – not detected/no data; hypothetical protein PJ17_07480 and hypothetical protein PJ17_07485 were actually a single protein with expression controlled by a homopolymeric tract, mis-annotated in the GenBank record used for proteomics analysis

Statistical analysis using Mann-Whitney test with Benjamini-Hochberg correction, 00-1597 vs the other three isolates: ^†^*P* <0.05, ^§^*P* <0.01, ^‡^*P* <0.001, **P* <0.0001
