# Supplementary material for: Comparison of genomes and proteomes of four whole genome-sequenced Campylobacter jejuni from different phylogenetic backgrounds
Source: PLoS One. 2018 Jan 2;13(1):e0190836. doi: 10.1371/journal.pone.0190836 (PMC5749857; doi:10.1371/journal.pone.0190836)
Supplement: S6 Table — (DOCX) [file pone.0190836.s017.docx]

S6 Table. Detection of proteins in the 00-6200 HS:4 CPB cluster using comparative 4-plex iTRAQ proteomic analysis.

| **Protein Identity** | **Non-exclusive peptides** | **Gene identity (LS-BSR)** | | | | **Protein average log_2_ fold change** | | | |
| --- | --- | --- | --- | --- | --- | --- | --- | --- | --- |
|  |  | **00-0949** | **01-1512** | **00-6200** | **00-1597** | **00-0949** | **01-1512** | **00-6200** | **00-1597** |
| *kpsS* capsule biosynthesis protein PJ18_07220^1^ | - | 1 | 1 | 0.99 | 0.88 | 0.01 | 0.04 | 1.39 | **2.25** |
|  | + |  |  |  |  | 0.07 | -0.02 | -0.31 | 0.43 |
| *kpsC* capsule biosynthesis protein PJ18_07225^1^ | - | 1 | 1 | 0.95 | 0.85 | -0.03 | -0.09 | **2.71** | -0.32 |
|  | + |  |  |  |  | -0.03 | -0.07 | 0.55 | 0.45 |
| adenylylsulfate kinase PJ18_07230 | - | 1 | 1 | 0.98 | 0 | 0.03 | 0.24 | **4.50**^†^ | 0.08 |
|  | + |  |  |  |  | 0.01 | 0.06 | **1.23**^†^ | -1.75 |
| sugar nucleotidyltransferase PJ18_07235 | - | 1 | 1 | 1 | 0.09 | **-0.02** | **0.25** | **0.57** | -3.69** |
|  | + |  |  |  |  | **-0.02** | **0.22** | **050** | -3.78** |
| glutamine amidotransferase PJ18_07240 | - | 1 | 1 | 1 | 0.11 | ND | ND | ND | ND |
|  | + |  |  |  |  | ND | ND | ND | ND |
| hypothetical protein PJ18_07245 | - | 1 | 1 | 0.99 | 0.02 | -0.42 | 0.80 | **5.29*** | 0.71 |
|  | + |  |  |  |  | 0.02 | 0.01 | **0.87*** | -2.42 |
| methyltransferase PJ18_07250 | - | 1 | 1 | 0.99 | 0.1 | 0.11 | **4.42**^‡‡^ | 1.47 | -0.58 |
|  | + |  |  |  |  | 0.12 | **4.47**^‡‡^ | 1.40 | -0.54 |
| methyltransferase PJ18_07255 | - | 1 | 1 | 0.98 | 0.06 | -0.03 | -0.19 | **5.46*** | -0.39 |
|  | + |  |  |  |  | 0.05 | -2.22 | **2.05*** | -2.95 |
| hypothetical protein PJ18_07260 | - | 1 | 1 | 0.44 | 0.17 | -0.26 | 0.39 | **4.64*** | 0.33 |
|  | + |  |  |  |  | -0.17 | 0.25 | **4.39*** | 0.18 |
| alpha-2,3-sialyltransferase PJ18_07265 | - | 0.13 | 0.13 | 1 | 0.23 | -0.24 | 0.00 | **4.45*** | 0.57 |
|  | + |  |  |  |  | -0.21 | -0.11 | **4.27*** | 0.66 |
| glycosyltransferase family 2 PJ18_07270 | - | 0.05 | 0.05 | 1 | 0.05 | -0.06 | 0.28 | **4.48*** | 0.34 |
|  | + |  |  |  |  | -0.07 | 0.25 | **4.44*** | 0.33 |
| capsular biosynthesis protein PJ18_07275 | - | 0 | 0 | 1 | 0 | ND | ND | ND | ND |
|  | + |  |  |  |  | ND | ND | ND | ND |
| uridine kinase PJ18_07280 | - | 0.04 | 0.04 | 1 | 0.04 | ND | ND | ND | ND |
|  | + |  |  |  |  | ND | ND | ND | ND |
| hypothetical protein PJ18_07285 | - | 0.09 | 0.09 | 1 | 0 | ND | ND | ND | ND |
|  | + |  |  |  |  | ND | ND | ND | ND |
| D-glycero-D-manno-heptose 1-phosphate guanosyltransferase PJ18_07290 | - | 1 | 1 | 0.91 | 0.29 | 0.02 | 0.12 | **3.60** | 0.27 |
|  | + |  |  |  |  | 0.01 | -0.11 | **1.17**^‡^ | -1.79 |
| phosphoheptose isomerase PJ18_07295 | - | 1 | 1 | 0.97 | 0.43 | -0.10 | -0.02 | **5.30**^‡^ | -0.20 |
|  | + |  |  |  |  | -0.03 | -0.24 | **1.45**^‡^ | -2.22 |
| dehydrogenase PJ18_07300 | - | 1 | 1 | 0.97 | 0.06 | 0.10 | 0.25 | **3.74**^§^ | 0.38 |
|  | + |  |  |  |  | **0.07** | **-0.10** | **-0.35** | -2.53** |
| membrane protein PJ18_07305^1^ | - | 1 | 1 | 0.98 | 0.12 | -0.12 | **1.35** | ND | ND |
|  | + |  |  |  |  | **-0.05** | **0.34** | **0.14** | -2.70** |
| GDP-mannose 4,6-dehydratase PJ18_07310 | - | 0.14 | 0.14 | 1 | 0.14 | ND | ND | ND | ND |
|  | + |  |  |  |  | ND | ND | ND | ND |
| GDP-4-keto-6-deoxy-D-mannose-3,5-epimerase-4-reductase PJ18_07315 | - | 1 | 1 | 0.59 | 0.06 | -0.08 | 0.36 | **4.47*** | 0.25 |
|  | + |  |  |  |  | -0.08 | 0.34 | **4.31*** | 0.11 |
| dDTP-4-dehydrorhamnose 3,5-epimerase PJ18_07320 | - | 1 | 1 | 0.81 | 0.19 | -0.27 | 0.51 | **5.24*** | 0.20 |
|  | + |  |  |  |  | -0.16 | 0.29 | **3.61*** | -0.80 |
| sugar transferase PJ18_07325 | - | 0.14 | 0.14 | 1 | 0.13 | -0.06 | 0.39 | **4.37*** | 0.38 |
|  | + |  |  |  |  | -0.07 | 0.28 | **4.24*** | 0.32 |
| hypothetical protein PJ18_07330 | - | 0.45 | 0.45 | 1 | 0.12 | -0.10 | 0.33 | **4.85*** | 0.16 |
|  | + |  |  |  |  | -0.06 | 0.07 | **4.36*** | -0.40 |
| capsular biosynthesis protein PJ18_07335 | - | 0.05 | 0.05 | 1 | 0.56 | 0.01 | 0.40 | **3.36**^§^ | -0.08 |
|  | + |  |  |  |  | 0.05 | 0.35 | **3.12**^§^ | 0.54 |
| sugar transferase PJ18_07340* | - | 1 | 1 | 0.97 | 0.07 | 0.00 | 0.26 | **3.06** | 0.57 |
|  | + |  |  |  |  | 0.00 | 0.02 | **0.84*** | -2.33 |
| *kpsF* arabinose-5-phosphate isomerase PJ18_07345 | - | 1 | 1 | 1 | 0.97 | **-0.01** | **-0.05** | **-0.12** | -4.02** |
|  | + |  |  |  |  | -0.01 | -0.07 | -0.08 | -1.68 |
| *kpsD* sugar ABC transporter substrate-binding protein PJ18_07350 | - | 1 | 1 | 1 | 0.99 | **0.00** | **0.07** | **0.10** | -2.20 |
|  | + |  |  |  |  | -0.33 | -0.22 | -0.13 | -0.21 |
| *kpsE* capsule biosynthesis protein PJ18_07355 | - | 1 | 1 | 0.98 | 1 | 0.34 | 0.32 | **4.51*** | 2.26 |
|  | + |  |  |  |  | -0.06 | 0.04 | 0.77 | 0.36 |
| *kpsT* ABC transporter ATP-binding protein PJ18_07360 | - | 1 | 1 | 1 | 0.98 | -0.01 | 0.23 | 0.45 | 0.43 |
|  | + |  |  |  |  | 0.00 | 0.21 | 0.40 | 0.42 |
| *kpsM* capsule biosynthesis protein PJ18_07365 | - | 1 | 1 | 0.97 | 0.97 | ND | ND | ND | ND |
|  | + |  |  |  |  | ND | ND | ND | ND |

Isolate 00-0949 was used as the reference strain for iTRAQ analysis except where otherwise noted; NP – not present; ND – not detected/no data; ^1^detected in only 1 or 2 replicate experiment when non-exclusive peptides were not used in the analysis

Statistical analysis using Mann-Whitney test with Benjamini-Hochberg correction, 00-6200 vs the other three isolates: ^†^*P* <0.05, ^§^*P* <0.01, ^‡^*P* <0.001, **P* <0.0001; 01-1512 vs the other three isolates: ^‡‡^*P* <0.001; 00-1597 vs the other three isolates: ***P* <0.0001
