# Supplementary material for: Comparison of genomes and proteomes of four whole genome-sequenced Campylobacter jejuni from different phylogenetic backgrounds
Source: PLoS One. 2018 Jan 2;13(1):e0190836. doi: 10.1371/journal.pone.0190836 (PMC5749857; doi:10.1371/journal.pone.0190836)
Supplement: S7 Table — (DOCX) [file pone.0190836.s018.docx]

S7 Table. Detection of proteins in the 00-0949 and 01-1512 HS:2 CPB cluster using comparative 4-plex iTRAQ proteomic analysis.

| **Protein Identity** | **Non-exclusive peptides** | **Gene identity (LS-BSR)** | | | | **Protein average log_2_ fold change** | | | |
| --- | --- | --- | --- | --- | --- | --- | --- | --- | --- |
|  |  | **00-0949** | **01-1512** | **00-6200** | **00-1597** | **00-0949** | **01-1512** | **00-6200** | **00-1597** |
| *kpsS* capsule biosynthesis protein PJ16_07800 | - | 1 | 1 | 0.99 | 0.88 | ND | ND | ND | ND |
|  | + |  |  |  |  | -0.01 | -0.12 | -0.72 | 0.07 |
| *kpsC* capsule biosynthesis protein PJ16_07805 | - | 1 | 1 | 0.95 | 0.85 | **-0.01** | **-0.05**^‡^ | -3.00 | -3.34 |
|  | + |  |  |  |  | **0.02** | **-0.06** | -1.21 | -1.76 |
| adenylylsulfate kinase PJ16_07810 | - | 1 | 1 | 0.98 | 0 | **0.08** | **0.16**^§^ | -3.95 | -4.49 |
|  | + |  |  |  |  | **0.01** | **0.09*** | -1.73 | -3.42 |
| sugar nucleotidyltransferase PJ16_07815 | - | 1 | 1 | 1 | 0.09 | **-0.02** | **0.25** | **0.57** | -3.69^#^ |
|  | + |  |  |  |  | **-0.02** | **0.22** | **050** | -3.78^#^ |
| glutamine amidotransferase PJ16_07820 | - | 1 | 1 | 1 | 0.11 | -0.01 | 0.02 | 0.71 | -2.28 |
|  | + |  |  |  |  | 0.05 | -0.03 | 0.28 | -2.99 |
| hypothetical protein PJ16_07825 | - | 1 | 1 | 0.99 | 0.02 | -0.42 | 0.80 | **5.29**^†^ | 0.71 |
|  | + |  |  |  |  | 0.02 | 0.01 | **0.87**^†^ | -2.42 |
| **methyltransferase PJ16_07830** | - | 1 | 1 | 0.99 | 0.1 | 0.11 | **4.42**^##^ | 1.47 | -0.58 |
|  | + |  |  |  |  | 0.12 | **4.47**^##^ | 1.40 | -0.54 |
| **methyltransferase PJ16_07835** | - | 1 | 1 | 0.98 | 0.06 | **0.26**** | -3.28 | -5.82 | -5.62 |
|  | + |  |  |  |  | **0.14**** | -3.45 | -1.43 | -4.86 |
| sugar transferase PJ16_07840 | - | 1 | 1 | 0.44 | 0.17 | **0.04**** | -3.03 | -4.47 | -4.37 |
|  | + |  |  |  |  | **0.04**** | -2.56 | -4.27 | -4.29 |
| sugar transferase PJ16_07845 | - | 1 | 1 | 0.45 | 0.12 | **-0.02** | **-0.01*** | -3.71 | -3.77 |
|  | + |  |  |  |  | **0.04** | **-0.26*** | -3.88 | -3.93 |
| D-glycero-D-manno-heptose 1-phosphate guanosyltransferase PJ16_07850 | - | 1 | 1 | 0.91 | 0.29 | **-0.05** | **0.50**^§^ | -3.76 | -4.57 |
|  | + |  |  |  |  | **0.03** | **0.40**^§^ | -2.42 | -3.85 |
| phosphoheptose isomerase PJ16_07855 | - | 1 | 1 | 0.97 | 0.43 | **-0.02** | **-0.15**^‡^ | -3.15 | -3.32 |
|  | + |  |  |  |  | **0.10** | **-0.08** | -0.62 | -3.59 |
| dehydrogenase PJ16_07860 | - | 1 | 1 | 0.97 | 0.06 | **0.02** | **-0.23*** | -3.42 | -3.25 |
|  | + |  |  |  |  | **-0.03** | **-0.19*** | -1.89 | -3.28 |
| hypothetical protein PJ16_07865 | - | 1 | 1 | 0.05 | 0.09 | **0.04** | **-0.98** | -4.97 | -4.77 |
|  | + |  |  |  |  | **0.05** | **-1.01** | -5.10 | -4.85 |
| membrane protein PJ16_07870 | - | 1 | 1 | 0.98 | 0.12 | **0.14** | **0.11*** | -5.00 | -4.77 |
|  | + |  |  |  |  | **0.10** | **0.03*** | -3.24 | -4.48 |
| GDP-4-keto-6-deoxy-D-mannose-3,5-epimerase-4-reductase PJ16_07875 | - | 1 | 1 | 0.59 | 0.06 | **0.02** | **-0.06*** | -3.85 | -3.93 |
|  | + |  |  |  |  | **0.01** | **-0.10*** | -3.90 | -4.02 |
| hypothetical protein PJ16_07880 | - | 1 | 1 | 0 | 0 | **0.11** | **-0.02*** | -5.15 | -4.96 |
|  | + |  |  |  |  | **0.13** | **-0.03*** | -5.31 | -5.06 |
| dDTP-4-dehydrorhamnose 3,5-epimerase PJ16_07885 | - | 1 | 1 | 0.81 | 0.19 | **0.07** | **-0.08*** | -4.10 | -4.61 |
|  | + |  |  |  |  | **0.07** | **0.10*** | -3.12 | -4.23 |
| capsule biosynthesis protein CapA PJ16_07890 | - | 1 | 1 | 0.18 | 0.11 | **0.03** | **0.02*** | -3.66 | -4.02 |
|  | + |  |  |  |  | **0.04** | **0.01*** | -3.78 | -4.08 |
| sugar transferase PJ16_07895 | - | 1 | 1 | 0.03 | 0.04 | **0.03** | **0.04*** | -3.72 | -3.68 |
|  | + |  |  |  |  | **0.03** | **0.02*** | -3.82 | -3.72 |
| UDP pyrophosphate phosphatase PJ16_07900 | - | 0.95 | 1 | 0.5 | 0.5 | **0.05** | **0.02*** | -3.64 | -3.50 |
|  | + |  |  |  |  | **0.03** | **-0.03*** | -3.82 | -3.59 |
| sugar transferase PJ16_07905 | - | 1 | 1 | 0.11 | 0.46 | **0.12** | **0.03*** | -4.96 | -4.42 |
|  | + |  |  |  |  | **0.08** | **-0.05*** | -4.75 | -4.36 |
| phosphatase PJ16_07910 | - | 1 | 1 | 0.11 | 0.11 | **0.01** | **-0.01*** | -3.75 | -3.47 |
|  | + |  |  |  |  | **0.03** | **-0.03*** | -3.84 | -3.47 |
| aminotransferase PJ16_07915 | - | 1 | 1 | 0.09 | 0.09 | **0.04** | **0.62**^‡^ | -1.93 | -2.09 |
|  | + |  |  |  |  | **0.04** | **0.63*** | -2.02 | -2.10 |
| aminotransferase PJ16_07920 | - | 1 | 1 | 0.14 | 0.14 | **-0.01** | **-0.11*** | -3.84 | -3.82 |
|  | + |  |  |  |  | **-0.01** | **-0.14*** | -3.97 | -3.88 |
| sugar transferase PJ16_07925 | - | 1 | 1 | 0.06 | 0.28 | **-0.05** | **0.03*** | -4.96 | -4.42 |
|  | + |  |  |  |  | **0.05** | **-0.10*** | -4.32 | -4.18 |
| UDP-galactopyranose mutase PJ16_07930 | - | 1 | 1 | 0 | 0.79 | **0.02** | **-0.17*** | -3.12 | -3.30 |
|  | + |  |  |  |  | **0.03** | **-0.19*** | -3.14 | -3.09 |
| glycosyltransferase PJ16_07935 | - | 1 | 1 | 0.43 | 0.45 | **-0.02** | **0.00*** | -2.64 | -2.67 |
|  | + |  |  |  |  | **0.01** | **-0.02*** | -2.54 | -2.55 |
| UDP-glucose-6-dehydrogenase PJ16_07940 | - | 1 | 1 | 0 | 0.15 | **0.02** | **0.00*** | -3.50 | -3.69 |
|  | + |  |  |  |  | **0.01** | **-0.03*** | -3.63 | -3.74 |
| sugar transferase PJ16_07945* | - | 1 | 1 | 0.97 | 0.07 | **-0.01** | **0.00** | **-**2.13 | -2.51 |
|  | + |  |  |  |  | -0.04 | -0.03* | **0.63** | -2.46 |
| *kpsF* arabinose-5-phosphate isomerase PJ18_07345 | - | 1 | 1 | 1 | 0.97 | **-0.01** | **-0.05** | **-0.12** | -4.02^††^ |
|  | + |  |  |  |  | **-0.01** | **-0.07** | **-0.08** | -1.68 |
| *kpsD* sugar ABC transporter substrate-binding protein PJ18_07350 | - | 1 | 1 | 1 | 0.99 | **0.00** | **0.07** | **0.10** | -2.20^††^ |
|  | + |  |  |  |  | -0.33 | -0.22 | -0.13 | -0.21 |
| *kpsE* capsule biosynthesis protein PJ16_07960 | - | 1 | 1 | 0.98 | 1 | **0.02** | **0.11** | -2.49^†^ | **0.37** |
|  | + |  |  |  |  | -0.01 | 0.07 | -0.82^†^ | -0.22 |
| *kpsT* ABC transporter ATP-binding protein PJ16_07965 | - | 1 | 1 | 1 | 0.98 | -0.01 | 0.23 | 0.45 | 0.43 |
|  | + |  |  |  |  | 0.00 | 0.21 | 0.40 | 0.42 |
| *kpsM* capsule biosynthesis protein PJ16_07970 | - | 1 | 1 | 0.97 | 0.97 | ND | ND | ND | ND |
|  | + |  |  |  |  | ND | ND | ND | ND |

Isolate 00-0949 was used as the reference strain for iTRAQ analysis except where otherwise noted; NP – not present; ND – not detected/no data

Statistical analysis using Mann-Whitney test with Benjamini-Hochberg correction, 00-0949 and 01-1512 vs 00-6200 and 00-1597: ^§^*P* <0.01, ^‡^*P* <0.001, **P* <0.0001; 01-1512 vs the other three isolates: ^##^*P* <0.001; 00-0949 vs the other three isolates: ***P* <0.0001; 00-6200 vs the other three isolates: ^†^*P* <0.0001; 00-1597 vs the other three isolates: ^#^*P* <0.0001;
