# Supplementary material for: Comparison of genomes and proteomes of four whole genome-sequenced Campylobacter jejuni from different phylogenetic backgrounds
Source: PLoS One. 2018 Jan 2;13(1):e0190836. doi: 10.1371/journal.pone.0190836 (PMC5749857; doi:10.1371/journal.pone.0190836)
Supplement: S8 Table — (DOCX) [file pone.0190836.s019.docx]

S8 Table. Detection of Transducer-like proteins (Tlps) using comparative 4-plex iTRAQ proteomic analysis.

| **Protein Identity** | **Non-exclusive peptides** | **Gene identity (LS-BSR)** | | | | **Protein average log_2_ fold change** | | | |
| --- | --- | --- | --- | --- | --- | --- | --- | --- | --- |
|  |  | **00-0949** | **01-1512** | **00-6200** | **00-1597** | **00-0949** | **01-1512** | **00-6200** | **00-1597** |
| chemotaxis protein Tlp1 (PJ18_07650)^1^ | - | 1 | 1 | 1 | 1 | ND | ND | ND | ND |
|  | + |  |  |  |  | 0.04 | 0.12 | -0.66 | -0.36 |
| chemotaxis protein Tlp1 (PJ16_08505; PJ19_08250)^1^ | - | 1 | 1 | 1 | 1 | **0.04** | **-0.04*** | -3.99 | ND |
|  | + |  |  |  |  | **0.03** | **0.08*** | -1.51 | -0.98 |
| chemotaxis protein Tlp3 (PJ17_08150) | - | 0.99 | 1 | 0.99 | 0.97 | ND | ND | ND | ND |
|  | + |  |  |  |  | 0.00 | -0.19 | 0.25 | 0.86 |
| chemotaxis protein Tlp3 (PJ18_00730) | - | 0.99 | 1 | 0.99 | 0.97 | **0.06** | **0.18*** | **0.63**^††^ | -3.53 |
|  | + |  |  |  |  | 0.08 | 0.38 | 0.58 | 0.19 |
| chemotaxis protein Tlp4 (PJ19_01250)^#^ | - | 0.52 | 1 | 0.56 | 0.75 | **0.00** | **1.24*** | -2.82 | -3.06 |
|  | + |  |  |  |  | -0.10 | 0.60 | -0.04 | 0.03 |
| chemotaxis protein Tlp11 (PJ18_01260) | - | 0.49 | 0.49 | 1 | 0.79 | -0.02 | 0.11 | **3.88**^†^ | 0.44 |
|  | + |  |  |  |  | 0.48 | 1.11 | 1.17 | 0.81 |
| chemotaxis protein Tlp12 (PJ17_01290) | - | 0.5 | 0.75 | 0.79 | 1 | -0.09 | -0.32 | 0.98 | **5.12**^§^ |
|  | + |  |  |  |  | 0.97 | 1.98 | 1.85 | **3.06**^§^ |
| chemotaxis protein Tlp13 (PJ17_01300) | - | 0.53 | 0.53 | 0.79 | 1 | -0.06 | -0.34 | 0.31 | **3.92**^§^ |
|  | + |  |  |  |  | 0.58 | 1.59 | 1.68 | **2.87**^§^ |
| chemotaxis protein Tlp14 (PJ17_00730) | - | 1 | 1 | 0.84 | 0.96 | -0.23 | -0.85 | 1.74 | **6.22**^§^ |
|  | + |  |  |  |  | -0.04 | -0.48 | -0.59 | **2.43**^§^ |
| chemotaxis protein Tlp14 (PJ19_00730) | - | 1 | 1 | 0.84 | 0.96 | **0.08** | **-0.99*** | -5.28 | -5.24 |
|  | + |  |  |  |  | **0.10** | **0.08*** | -2.74 | -3.55 |

Isolate 00-0949 was used as the reference strain for iTRAQ analysis; NP – not present; ND – not detected/no data; ^1^LS-BSR analysis indicated complete identity, but the predicted proteins were annotated as different in the different isolates; ^#^gene was disrupted in isolate 00-0949

Statistical analysis using Mann-Whitney test with Benjamini-Hochberg correction, 00-0949 and 01-1512 vs 00-6200 and 00-1597: **P* <0.0001; 00-6200 vs the other three isolates: ^†^*P* <0.0001; 00-6200 vs the other three isolates: ^†^*P* <0.0001; 00-6200 vs 00-1597: ^††^*P* <0.0001; 00-1597vs the other three isolates, ^§^*P* <0.0001
