# Supplementary material for: Comparison of genomes and proteomes of four whole genome-sequenced Campylobacter jejuni from different phylogenetic backgrounds
Source: PLoS One. 2018 Jan 2;13(1):e0190836. doi: 10.1371/journal.pone.0190836 (PMC5749857; doi:10.1371/journal.pone.0190836)
Supplement: S9 Table — (DOCX) [file pone.0190836.s020.docx]

S9 Table. Detection of selected iron acquisition and oxidative stress proteins in the four *C. jejuni* isolates using comparative iTRAQ proteomic analysis.

| **Protein Identity (locus tags in selected isolates)** | **Non-exclusive peptides** | **Gene identity (LS-BSR)** | | | | **Protein average log_2_ fold change** | | | |
| --- | --- | --- | --- | --- | --- | --- | --- | --- | --- |
|  |  | **00-0949** | **01-1512** | **00-6200** | **00-1597** | **00-0949** | **01-1512** | **00-6200** | **00-1597** |
| biopolymer transporter ExbB (PJ16_00900; PJ17_00900) | - | 1 | 1 | 1 | 0.96 | -0.12 | -0.04 | 1.27 | **2.71**^†^ |
|  | + |  |  |  |  | -0.14 | -0.07 | 1.21 | **2.79**^†^ |
| biopolymer transporter ExbD (PJ17_00560)^1^ | - | 1 | 1 | 1 | 1^2^ | 0.01 | 0.44 | **2.07** | **2.43** |
|  | + |  |  |  |  | -0.01 | 0.15 | 0.28 | 0.53 |
| biopolymer transporter ExbD (PJ19_00560) | - | 1 | 1 | 1 | 1^2^ | **-0.07** | **0.33** | -3.15 | -3.10^††^ |
|  | + |  |  |  |  | -0.04 | 0.11 | -0.46 | 0.24 |
| biopolymer transporter ExbD (PJ16_08850; PJ17_08495) ^1^ | - | 1 | 1 | 0.99 | 0.99 | ND | ND | ND | ND |
| 01-1512 used as reference | + |  |  |  |  | 1.68 | 0.69 | 2.28 | **6.75**^‡^ |
| catalase (PJ17_07315) | - | 1 | 1 | 1 | 1^2^ | -0.01 | -0.83 | 1.53 | **3.96**^‡^ |
|  | + |  |  |  |  | -0.08 | -0.32 | 1.57 | **4.12*** |
| catalase (PJ16_07665)^1^ | - | 1 | 1 | 1 | 1^2^ | **0.01** | **0.34** | -1.97 | -1.96 |
|  | + |  |  |  |  | -0.07 | -0.22 | 1.55 | **4.06*** |
| ferric enterobactin uptake receptor CfrA(PJ17_03645) | - | 1 | 1 | 1 | 0.98 | -0.13 | -0.37 | 0.87 | **4.48*** |
| 01-1512 used as reference | + |  |  |  |  | -0.22 | -0.09 | 075 | **4.11*** |
| ferric enterobactin uptake receptor CfrA (PJ16_04040) | - | 1 | 1 | 1 | 0.98 | **0.02** | **0.45** | **0.41** | -1.05 |
|  | + |  |  |  |  | -0.05 | 0.39 | 0.83 | **3.68*** |
| ferrous iron transporter A (PJ17_06525) 01-1512 used as reference | - | 1 | 1 | 0.99 | 0.99 | -0.06 | 0.01 | 0.11 | 0.11 |
|  | + |  |  |  |  | 0.00 | 0.06 | 0.17 | 0.16 |
| ferrous iron transporter A (PJ18_06330) 01-1512 used as reference | - | 1 | 1 | 0.99 | 0.99 | -0.07 | 0.00 | 0.12 | 0.06 |
|  | + |  |  |  |  | 0.01 | 0.07 | 0.20 | 0.14 |
| ferrous iron transporter A (PJ19_07170) | - | 1 | 1 | 0.99 | 0.99 | **0.22** | **0.49** | -1.30 | **-**0.99^†^ |
|  | + |  |  |  |  | -0.01 | 0.12 | 0.08 | 0.05 |
| ferrous iron transporter A (PJ17_07125) 01-1512 used as reference | - | 1 | 1 | 0.97 | 0.98 | 0.15 | 0.02 | 0.44 | -0.13 |
|  | + |  |  |  |  | -0.03 | -0.10 | 0.32 | -0.25 |
| ferrous iron transporter A (PJ18_06925, PJ16_07485)^1^ 01-1512 used as reference | - | 1 | 1 | 0.97 | 0.98 | 0.15 | 0.02 | 0.44 | -0.13 |
|  | + |  |  |  |  | -0.03 | -0.10 | 0.32 | -0.25 |
| hemin transporter ChuD (PJ17_08415) | - | 1 | 1 | 0.99 | 0.99 | 0.45 | -0.61 | 0.19 | **3.89**^‡^ |
|  | + |  |  |  |  | -0.03 | -0.05 | 0.39 | **3.37*** |
| hemin transporter ChuD (PJ18_08170; PJ16_8770; PJ19_9760) | - | 1 | 1 | 0.99 | 0.99 | ND | ND | ND | ND |
|  | + |  |  |  |  | -0.04 | 0.15 | 0.73 | **3.19*** |
| iron ABC transporter ATP-binding protein CfbpC (PJ17_00870) | - | 1 | 1 | 1 | 0.96 | 0.23 | -0.38 | 0.53 | **3.82**^‡^ |
|  | + |  |  |  |  | 0.02 | -0.12 | 0.18 | **2.28*** |
| iron ABC transporter ATP-binding protein CfbpC (PJ16_00870) | - | 1 | 1 | 1 | 0.96 | 0.14 | -0.38 | 0.64 | **-2.22**^‡^ |
|  | + |  |  |  |  | 0.03 | 0.09 | 0.36 | **1.47*** |
| iron deficiency-induced protein A CfbpA (PJ17_00880) | - | 1 | 1 | 1 | 1^2^ | -0.16 | -0.58 | 0.98 | **4.06**^§^ |
|  | + |  |  |  |  | -0.07 | 0.00 | 0.70 | **2.86*** |
| iron deficiency-induced protein A CfbpA (PJ16_00880) | - | 1 | 1 | 1 | 1^2^ | **0.01** | **0.28** | **0.69** | -2.06^§^ |
|  | + |  |  |  |  | -0.07 | 0.11 | 0.36 | **1.47*** |
| iron permease (PJ17_08640) | - | 1 | 1 | 0.99 | 0.99 | 0.00 | -0.74 | 0.18 | **0.97** |
|  | + |  |  |  |  | -0.01 | 0.12 | 0.07 | **1.79*** |
| iron permease (PJ18_08390) | - | 1 | 1 | 0.99 | 0.99 | -0.03 | 0.15 | **5.32** | 0.87^§§^ |
|  | + |  |  |  |  | 0.00 | 0.27 | **1.13** | **1.52**** |
| iron permease (PJ16_08990; PJ19_08980) 01-1512 used as reference | - | 1 | 1 | 0.99 | 0.99 | -0.31 | -0.02 | -0.17 | **1.49** |
|  | + |  |  |  |  | -0.01 | 0.27 | 0.12 | **1.78*** |
| iron transporter (PJ17_00890) | - | 1 | 1 | 1 | 1 | 0.17 | 0.37 | 0.76 | **3.26*** |
|  | + |  |  |  |  | -0.19 | 0.35 | 0.68 | **3.26*** |
| iron transporter (PJ17_08645) | - | 1 | 1 | 1 | 0.99 | -0.15 | 0.27 | 0.81 | **2.71*** |
|  | + |  |  |  |  | -0.14 | 0.26 | 0.76 | **2.73*** |
| iron-binding protein (PJ17_00250) | - | 1 | 1 | 0.98 | 1 | -0.11 | **0.63** | -1.18 | -0.50 |
|  | + |  |  |  |  | **-0.03** | **0.33** | -1.70 | -0.97 |
| iron-binding protein (PJ18_00250) | - | 1 | 1 | 0.98 | 1 | ND | ND | ND | ND |
|  | + |  |  |  |  | 0.00 | 0.31 | -0.49 | -1.23* |
| peroxidase (PJ17_01620) | - | 1 | 1 | 1 | 1 | -0.03 | -0.04 | 0.67 | **1.42*** |
|  | + |  |  |  |  | -0.03 | -0.09 | 0.58 | **1.41*** |
| peroxiredoxin (PJ17_01330) | - | 1 | 1 | 1 | 0.98 | 0.90 | -0.55 | 0.25 | **3.68**^§^ |
|  | + |  |  |  |  | 0.03 | -0.23 | 0.06 | **0.94**^†^ |
| peroxiredoxin (PJ18_01305; PJ16_01295; PJ19_01295) | - | 1 | 1 | 1 | 0.98 | **0.01** | **0.19** | **0.24** | -1.85* |
|  | + |  |  |  |  | **-0.01** | **0.04** | **0.17** | -0.70 |
| thioredoxin 11 kDa (PJ17_00745; PJ16_00745; PJ19_00745) | - | 1 | 1 | 0.81 | 0.97 | **0.02** | **0.03** | -2.50^§§^ | **0.26** |
|  | + |  |  |  |  | **0.02** | **0.00** | -0.83^#^ | **022** |
| thioredoxin 11 kDa (PJ18_01245) | - | 1 | 1 | 0.81 | 0.97 | 0.15 | 0.33 | **5.64**^§§^ | 0.52 |
|  | + |  |  |  |  | 0.02 | 0.03 | 0.55 | 0.29 |
| thioredoxin 18 kDa (PJ18_08420; PJ17_08670; PJ16_09020; PJ19_09010) | - | 1 | 1 | 1 | 1 | 0.00 | 0.15 | 0.31 | **1.66*** |
|  | + |  |  |  |  | -0.01 | 0.09 | 0.23 | **1.65*** |
| thioredoxin 21 kDa (PJ17_06165; PJ16_06540; PJ19_06805; PJ18_05965) | - | 1 | 1 | 1 | 0.98 | -0.05 | 0.12 | 0.25 | 0.66^‡^ |
|  | + |  |  |  |  | -0.06 | 0.07 | 0.14 | 0.63* |
| thioredoxin 23 kDa (PJ17_05675) | - | 1 | 1 | 1 | 0.99 | 0.15 | -1.32 | 1.20 | **5.76**^§^ |
|  | + |  |  |  |  | 0.00 | -0.20 | 0.49 | **0.76**^†^ |
| thioredoxin 23 kDa (PJ18_05485 ; PJ16_05770; PJ19_06035) | - | 1 | 1 | 1 | 0.99 | **0.10** | **-0.34** | **0.06** | -2.13^†^ |
|  | + |  |  |  |  | 0.01 | -0.08 | 0.43 | -0.10 |
| TonB-dependent receptor (PJ18_01095) | - | 1 | 1 | 1 | 0.99 | 0.23 | 0.47 | 1.22 | **2.88*** |
|  | + |  |  |  |  | 0.19 | 0.40 | 1.14 | **2.88*** |

Isolate 00-0949 was used as the reference strain for iTRAQ analysis except where noted otherwise; ND – not detected; ^1^detected in only one replicate experiment when non-exclusive peptides were not included in the analysis; ^2^LS-BSR value for 00-01597 should be <1 since annotated proteins were not completely identical

Statistical analysis using Mann-Whitney test with Benjamini-Hochberg correction; 00-1597 vs the other three isolates: ^†^*P* <0.05, ^§^*P* <0.01, ^‡^*P* <0.001, **P* <0.0001; 01-1512 vs the other three isolates: ^‡‡^*P* <0.001; 00-0949 and 00-1512 vs 00-6200 and 00-1597: ^††^*P* <0.05, ***P* <0.0001; 00-6200 vs the other three isolates: ^§§^*P* <0.01, ^#^*P* <0.0001
