# Supplementary material for: Comparison of genomes and proteomes of four whole genome-sequenced Campylobacter jejuni from different phylogenetic backgrounds
Source: PLoS One. 2018 Jan 2;13(1):e0190836. doi: 10.1371/journal.pone.0190836 (PMC5749857; doi:10.1371/journal.pone.0190836)
Supplement: S10 Table — (DOCX) [file pone.0190836.s021.docx]

S10 Table. Selected proteins exhibiting differential detection in the four *C. jejuni* isolates using comparative iTRAQ proteomic analysis.

| **Protein Identity (locus tags in selected isolates)** | **Non-exclusive peptides** | **Gene identity (LS-BSR)** | | | | **Protein average log_2_ fold change** | | | |
| --- | --- | --- | --- | --- | --- | --- | --- | --- | --- |
|  |  | **00-0949** | **01-1512** | **00-6200** | **00-1597** | **00-0949** | **01-1512** | **00-6200** | **00-1597** |
| ABC transporter substrate-binding protein (PJ18_02065) | - | 1 | 1 | 1 | 0 | -0.09 | 0.15 | **1.85**^#^ | -1.68* |
|  | + |  |  |  |  | -0.07 | 0.15 | **1.79**^#^ | -1.78* |
| acetylornithine aminotransferase (PJ18_00900) | - | 1 | 1 | 1 | 0.99 | 0.02 | 0.07 | **-1.77**^#^ | **-0.77*** |
|  | + |  |  |  |  | 0.01 | 0.03 | **-1.89**^#^ | **-0.80*** |
| altronate hydrolase 42 kDa (PJ18_02405) | - | 1 | 1 | 1 | 0 | -0.01 | 0.22 | **1.30**^#^ | -1.14^§^ |
|  | + |  |  |  |  | 0.00 | 0.20 | **1.24**^#^ | -1.15* |
| aspartate ammonia-lyase (PJ17_00445) | - | 1 | 1 | 1 | 1 | -0.01 | -0.12 | **1.37**^#^ | 0.42 |
|  | + |  |  |  |  | -0.01 | -0.14 | **1.30**^#^ | 0.43 |
| beta lactamase (PJ18_01430) | - | 1 | 1 | 1 | 0 | 0.08 | **3.06**^‡‡^ | -0.46 | -0.58 |
|  | + |  |  |  |  | 0.08 | **3.14**^‡‡^ | -0.50 | -0.69 |
| dihydroneopterin aldolase (PJ16_00085) | - | 1 | 1 | 1 | 1 | 0.01 | 0.35 | **-1.95**^#^ | -0.19 |
|  | + |  |  |  |  | 0.02 | 0.34 | **-2.07**^#^ | -0.19 |
| dihydropicolinate synthase (PJ18_02395) | - | 1 | 1 | 1 | 0.15 | 0.01 | 0.16 | **1.48**^#^ | -2.25* |
|  | + |  |  |  |  | -0.01 | 0.12 | **1.42**^#^ | **-**2.34* |
| ferritin (PJ17_02970) | - | 1 | 1 | 1 | 1 | -0.08 | 0.04 | **2.60**^#^ | **1.89*** |
|  | + |  |  |  |  | -0.10 | -0.04 | **2.48**^#^ | **1.83*** |
| flagellar basal body rod protein FlgC (PJ17_02550) | - | 1 | 1 | 1 | 1 | 0.00 | 0.32 | 0.12 | 0.27 |
|  | + |  |  |  |  | -0.05 | -0.05** | **1.61** | **1.10** |
| heavy metal transport/detoxification protein (PJ17_05940) | - | 1 | 1 | 1 | 1 | -0.05 | 0.06 | 0.05 | **1.73*** |
|  | + |  |  |  |  | -0.05 | 0.03 | -0.04 | **1.75*** |
| hypothetical protein (PJ16_06080, PJ17_05975) | - | 1 | 1 | 1 | 1 | **-0.05** | **0.36** | -1.63 | -0.93 |
|  | + |  |  |  |  | **0.08** | **0.44^††^** | -1.68 | -0.94 |
| hypothetical protein (PJ16_06720, PJ17_06345) | - | 1 | 1 | 1 | 1 | 0.00 | 0.07 | -0.02 | **-1.36** |
|  | + |  |  |  |  | -0.02 | 0.04 | -0.13 | **-1.40** |
| lipoprotein 120 KDa (PJ16_03130, PJ19_03395) | - | ND | ND | ND | ND | 0.07 | **3.77**^##^ | 0.92 | 0.07 |
|  | + |  |  |  |  | -0.01 | **4.18**^‡‡^ | -0.09 | 0.15 |
| lipoprotein 120 KDa (PJ17_03045) | - | ND | ND | ND | ND | 0.01 | -0.14 | 0.41 | **3.65** |
|  | + |  |  |  |  | -0.02 | **4.00**^‡‡^ | 0.15 | 0.27 |
| lipoprotein 93 kDa (PJ16_09115, PJ19_09105, PJ18_08515) | -^1^ | 1 | 1 | 0.98 | 0.90 | 0.08 | 0.31 | ND | ND |
|  | + |  |  |  |  | 0.01 | **4.34**^‡‡^ | -0.15 | 0.10 |
| membrane protein (PJ17_04470) | - | 1 | 1 | 1 | 1 | -0.05^+^ | **2.58** | **2.53** | **2.06** |
|  | + |  |  |  |  | -0.04^+^ | **2.58** | **2.47** | **2.08** |
| molybdenum cofactor biosynthesis protein MoaA (PJ17_07945) | - | 1 | 1 | 1 | 0.98 | -0.06 | -0.25 | 1.04 | **4.75*** |
|  | + |  |  |  |  | -0.03 | 0.24 | 0.84 | **3.03*** |
| molybdenum cofactor biosynthesis protein MoaA (PJ19_08315) | - | 1 | 1 | 1 | 0.98 | **-0.02** | **0.37** | **0.11** | -2.48^‡^ |
|  | + |  |  |  |  | -0.01 | -0.04 | 0.64 | **1.72*** |
| molybdenum cofactor biosynthesis protein MoaD (PJ17_07930) | - | 1 | 1 | 0.99 | 0.99 | 0.00 | 0.13 | 0.65 | **1.96*** |
|  | + |  |  |  |  | -0.02 | 0.10 | 0.56 | **1.95*** |
| molybdenum cofactor biosynthesis protein MoaE (PJ17_07935) | - | 1 | 1 | 1 | 0.98 | 0.10 | -0.47 | 0.51 | **3.24**^‡^ |
|  | + |  |  |  |  | 0.04 | -0.29 | 0.37 | **2.40*** |
| molybdenum cofactor biosynthesis protein MoaE (PJ16_08315) | - | 1 | 1 | 1 | 0.98 | 0.02 | 0.04 | 0.44 | -2.61^‡^ |
|  | + |  |  |  |  | -0.09 | -0.04 | 0.27 | **1.66*** |
| nitroreductase (PJ17_05475) | - | 1 | 1 | 1 | 0.97 | ND | ND | ND | ND |
|  | + |  |  |  |  | -0.01 | -0.21 | **2.93**^#^ | -1.10 |
| nitroreductase (PJ18_05275) | - | 1 | 1 | 1 | 0.97 | 0.02 | 0.02 | **2.74**^#^ | -1.48 |
|  | + |  |  |  |  | 0.01 | -0.12 | **2.78**^#^ | -1.25 |
| oxidoreductase 27 kDa (PJ17_02015) | - | 1 | 1 | 1 | 0.99 | -0.48 | -0.36^††^ | 0.87 | **3.95** |
|  | + |  |  |  |  | -0.26 | -0.29^++^ | **1.39** | **1.80** |
| oxidoreductase 27 kDa (PJ18_02015) | - | 1 | 1 | 1 | 0.99 | 0.00 | 0.04^††^ | **1.29** | -1.76 |
|  | + |  |  |  |  | -0.03 | -0.12^++^ | **1.47** | **0.78** |
| proline:sodium symporter PutP (PJ17_07855) | - | 1 | 1 | 1 | 1 | -0.03 | -0.26 | -0.17 | **0.82**^‡^ |
|  | + |  |  |  |  | -0.02 | -0.28 | -0.24 | **0.97**^‡^ |
| short-chain dehydrogenase (PJ18_02415) | - | 1 | 1 | 1 | 0.06 | -0.02 | 0.08 | **1.62**^#^ | -2.34 |
|  | + |  |  |  |  | -0.01 | 0.08 | **1.57**^#^ | -2.38 |
| trimethylamine N-oxide reductase I catalytic subunit (PJ18_01270) | - | 1 | 1 | 0.99 | 0.04 | 0.12 | 0.71 | **4.04**^§§^ | 0.57 |
|  | + |  |  |  |  | -0.08 | 0.56 | **3.72**^#^ | -0.77 |
| trimethylamine N-oxide reductase I catalytic subunit (PJ19_01260) | - | 1 | 1 | 0.99 | 0.04 | 0.01 | -0.33 | ND | -4.36 |
|  | + |  |  |  |  | -0.08 | 0.47 | **3.39**^#^ | -1.12 |

Isolate 00-0949 was used as the reference strain for iTRAQ analysis except where noted otherwise; ND – not detected; ^1^detected in only one replicate experiment when non-exclusive peptides were not included in the analysis. Statistical analysis using Mann-Whitney test with Benjamini-Hochberg correction, 00-1597 vs the other three isolates: ^†^*P* <0.05, ^§^*P* <0.01, ^‡^*P* <0.001, **P* <0.0001; 00-0949 and 00-1512 vs 00-6200 and 00-1597: ^††^*P* <0.01, ***P* <0.001, ^++^*P* <0.0001; 00-0949 vs the other three isolates, *P* <0.01, ^+^*P* <0.0001; 01-1512 vs the other three isolates: ^##^*P* <0.01, ^‡‡^*P* <0.0001; 00-6200 vs the other three isolates, ^§§^*P* <0.01, ^#^*P* <0.0001
